# Nebulized Aerosol Characteristics During Nasal High-Flow: Effect of Flow, Formulation and Impactor Temperature

**DOI:** 10.3390/pharmaceutics18101234

**Published:** 2026-09-29

**Authors:** Sasi Bhushan Yarragudi, Christina Koshy Vallipurayidathil, Andrew R. Martin, Stanislav Tatkov

**Affiliations:** 1 Fisher & Paykel Healthcare Ltd., Auckland 2013, New Zealand; 2 Mechanical Engineering and Biomedical Engineering, University of Alberta, Edmonton, AB T6G 1H9, Canada

**Keywords:** nasal high flow (NHF), high-flow nasal cannula (HFNC), high-flow oxygen therapy (HFOT), aerosol characterization, trans nasal aerosol delivery, NHF delivery efficiency, aerodynamic particle size distribution, nebulization, hygroscopic aerosols

## Abstract

**Background:** Aerosol delivery during nasal high-flow (NHF) therapy occurs within a heated and humidified environment where formulation composition, NHF rate, and Next Generation Impactor (NGI)/system temperature may jointly influence aerosol behavior. However, their combined effects following NHF-conditioned transport remain incompletely characterized. **Methods:** A balanced 4 × 2 × 2 full-factorial design evaluated NGI/system temperature (5, 21, 37, and 40 °C), formulation (isotonic and 3% hypertonic salbutamol formulations), and NHF rate (30 and 50 L·min^−1^). Aerosols generated using a vibrating mesh nebulizer integrated within an NHF system were transported through an adult nasal replica coupled to an NGI. Particle-size, fine-particle, delivery-efficiency, and regional-deposition metrics were analyzed using factorial ANOVA. **Results:** NHF rate exerted the greatest influence on downstream aerosol delivery, with increasing NHF rate reducing delivery efficiency, Fine Particle Dose (FPD), and NGI deposition while increasing NHF circuit retention. Significant Temperature × Formulation × Flow interactions were observed for MMAD (*p* = 0.0055), D16 (*p* < 0.0001), GSD (*p* = 0.0009), Fine Particle Fraction (FPF) (*p* = 0.0042), and dose-normalized FPD (*p* = 0.0149). The hypertonic formulation exhibited greater thermal sensitivity across several particle metrics. Although increasing NGI/system temperature generally reduced MMAD, D16, and D84, similar reductions in MMAD frequently produced markedly different responses in FPF, FPD, delivery efficiency, and regional deposition. All aerosols remained within ranges commonly associated with pulmonary deposition. **Conclusions:** Aerosol characteristics during NHF are governed by interacting effects of flow, formulation, and NGI/system temperature. Measurements conducted at NGI/system temperatures approaching body temperature provide additional mechanistic insight, particularly for hypertonic aerosols, and support integrated assessment of particle-size, delivery, and deposition outcomes rather than APSD metrics alone.

## 1. Introduction

Nasal high-flow (NHF) or high flow nasal cannula (HFNC) therapy is a non-invasive respiratory support modality that delivers heated and humidified gases at flow rates capable of meeting or exceeding a patient’s inspiratory demand. Initially introduced as an alternative to conventional oxygen interfaces, NHF has become increasingly prominent by demonstrating improved oxygenation, reduced need for escalation to invasive mechanical ventilation, and superior patient tolerance [1]. Subsequent studies in peri- and post-extubation proved NHF to be as effective as mask-based non-invasive ventilation in both high-risk and low-risk populations [2,3]. Most recently, large multicenter randomized trials and international clinical practice guidelines have established NHF as a first-line respiratory support modality across a range of acute respiratory failure phenotypes [4,5].

As NHF has become established as a first-line respiratory support strategy, interest in concurrent aerosol therapy has increased because inhaled medicines can be administered without interrupting respiratory support, particularly in patients unable to tolerate device removal [6,7,8]. Under NHF conditions, the aerosol traverses a heated and humidified high-flow pathway—including the humidification chamber, tubing, and nasal cannula—during which its physical properties may evolve from initial values at the point of generation. Droplet size evolves continuously during transport in response to humidity, temperature, and formulation composition. Experimental and scintigraphy studies consistently demonstrate that nebulizer type, circuit placement, NHF rate, and device platform substantially influence delivery efficiency [6,7,9,10,11,12,13,14]. Across systems, increasing flow reduces delivered dose, and the aerosol emerging downstream often appears finer owing to upstream loss of larger droplets through impaction in the flow circuit [11,12,13,14,15,16,17]. Hygroscopic growth studies further confirm that medical aerosols can undergo size changes when relative humidity approaches physiological conditions, reinforcing that the aerodynamic size distribution of an inhaled drug product is often a transient property shaped by the delivery environment rather than a fixed nebulizer output [18,19,20]. These hygroscopic size changes, coupled with pathway-dependent losses, therefore add complexity to the characterization of aerosol administration concurrent with NHF.

Because aerosol state evolves during transport, its characterization under NHF delivery conditions is environment-dependent. The Next Generation Impactor (NGI) is the dominant in vitro tool for aerodynamic particle-size distribution (APSD) assessment; however, aqueous aerosols are susceptible to evaporation or condensation artifacts if impactor temperature is mismatched to the conditioned aerosol stream. Studies of refrigerated cascade impactor operation demonstrate that impactor temperature and volumetric flow must be treated as coupled components of the measurement environment rather than passive background conditions [21,22]. Accordingly, APSD measured under NHF conditions reflects the combined interaction between the thermal state of the aerosol, the delivery pathway, and the impactor system, rather than nebulizer output alone.

These combined effects are particularly relevant for hygroscopic, high-tonicity formulations such as hypertonic saline, which may exhibit increased responsiveness to humidified environments. Hypertonic saline-based aerosols are clinically important in cystic fibrosis, bronchiectasis and bronchiolitis because their therapeutic effect depends on delivered osmotic activity, which promotes airway hydration, mucus mobilization, and mucociliary clearance [23,24,25,26,27]. Under the heated, humidified, high-flow conditions imposed by NHF, tonicity therefore represents a critical determinant of aerosol behavior rather than a passive formulation attribute.

Within this framework, extensive prior studies have established that NHF, formulation properties, and delivery-pathway characteristics each influence aerosol delivery during NHF therapy, and that aqueous pharmaceutical aerosols are inherently hygroscopic and formulation dependent [6,7,8,9,10,11,12,13,14,15,16,18,19]. Computational and experimental studies further indicate that under NHF conditions, formulation-dependent aerosol growth and system losses may decouple particle size from delivered lung dose [28]. However, although the individual effects of flow, formulation, and delivery-pathway conditions are increasingly recognized, how these factors interact under clinically relevant NHF conditions remains poorly understood. Most published studies have examined variables such as flow rate, device platform, or formulation in isolation. Consequently, limited information is available on aerosol behavior when transport through a conditioned NHF pathway, downstream thermal conditions, and formulation-dependent hygroscopic responses occur simultaneously [6,8,9,11,12,13,14,15,16,21]. This knowledge gap is important because changes in aerodynamic particle-size descriptors do not necessarily translate directly into equivalent changes in fine-particle delivery, delivered dose, or regional deposition. Therefore, aerosol characteristics under NHF cannot be inferred from particle-size measurements alone, and the relationship between aerosol state and aerosol delivery remains incompletely characterized [6,8,9,11,12,13,14,15,16].

Accordingly, this study, using isotonic and hypertonic salbutamol formulations, aimed to determine how formulation composition, NHF, and downstream thermal conditions jointly shape aerosol characteristics. Aerosol behavior was evaluated across a range of NGI/system temperatures and two clinically relevant NHF rates. We hypothesized that (i) formulation composition, NHF, and NGI/System temperature would interact to influence aerosol characteristics following NHF-conditioned transport (ii) these interaction effects would be more pronounced for the hypertonic formulation, reflecting greater hygroscopic sensitivity to NHF-conditioned transport; and (iii) changes in aerodynamic particle-size descriptors would not necessarily translate into equivalent changes in fine-particle delivery, delivered dose, or deposition behavior.

## 2. Materials and Methods

### 2.1. Materials and Formulations

Commercial isotonic salbutamol sulfate (ISS) solution (Asthalin^®^, Cipla Ltd., Mumbai, India, 5 mg per 2.5 mL unit-dose vial) was used throughout the study. The 3% hypertonic salbutamol sulfate (3% HSS) formulation was prepared by appropriately diluting a sterile 7% hypertonic saline solution together with the commercial salbutamol formulation to obtain a final solution containing 3% NaCl and salbutamol sulfate at 1 mg mL^−1^. The nominal loaded dose of salbutamol sulfate per run was 5 mg for the ISS formulation and 2.5 mg for the 3% HSS formulation.

### 2.2. Experimental Setup

A vibrating mesh nebulizer (Aerogen Solo, Aerogen Ltd., Galway, Ireland) integrated into the NHF circuit was used for aerosol generation. NHF, comprising heated (37 °C) and fully humidified (100% relative humidity) air, was delivered at two high flow rates (30 or 50 L·min^−1^) using an Airvo™ 3 system (Fisher & Paykel Healthcare, Auckland, New Zealand). An Optiflow™ DUET large-size asymmetric nasal cannula was used for all experiments. The Airvo device and nebulizer were positioned outside the environmental chamber throughout all experiments. The nasal cannula was connected to a custom 3D-printed adult nasal replica, which was coupled directly to a Next Generation Impactor (NGI; Copley Scientific Ltd., Nottingham, UK). A calibrated vacuum pump (Copley Scientific Ltd.) was connected downstream of the NGI. The nasal replica and NGI were housed inside a temperature- and humidity-controlled environmental chamber (Vötsch Industrietechnik GmbH, Balingen, Germany). A schematic of the experimental setup is provided in Figure 1.

The nasal replica was based on an averaged adult European upper-airway geometry truncated at the nasopharynx with a total volume of 30.2 mL and a wall surface area of 194.4 cm^2^. The geometry, airflow behavior, and relevance to asymmetric NHF ventilation have been described previously [29]. The nasal replica was 3D printed using VisiJet^®^ M3 clear material(3D Systems, Rock Hill, SC, USA), resulting in a smooth internal surface without any coating. The asymmetric nasal cannula was inserted into the nostrils in a consistent orientation for all experiments. The environmental chamber was maintained at a constant relative humidity of 50% for all experimental conditions. Chamber temperature stabilized at 5, 21, 37, or 40 °C prior to each run and was maintained throughout aerosol delivery and collection. These temperatures refer to the environmental chamber set point surrounding the nasal replica and NGI. Aerosol temperature, local humidity, and component temperatures were not directly measured during the experiments.

### 2.3. NGI Operation and Flow Measurement

The NGI was operated at volumetric flow rates matching the NHF rates under investigation (30 or 50 L·min^−1^). Before each run, volumetric flow at the NGI inlet was adjusted and verified using a calibrated Alicat mass-flow measurement device (Alicat Scientific, Tucson, AZ, USA). The NGI, vacuum pump, and NHF system were operated continuously for at least 10 min prior to nebulization to ensure stabilization of volumetric flow, temperature, and system conditions. For NGI operation at 50 L·min^−1^, which is not a pharmacopeial calibration flow for nebulizer testing, aerodynamic NGI stage cut-off diameters at 50 L min^−1^ were calculated using the stage-specific exponents from the published archival NGI calibration model [30,31,32]. The governing equation and corresponding stage-specific cut-off diameters are provided in Supplementary Material S1.

### 2.4. Experimental Design

A balanced 4 × 2 × 2 full-factorial design was employed. The fixed factors were NGI/system temperature (5, 21, 37, and 40 °C), formulation (ISS or 3% HSS), and NHF rate (30 or 50 L·min^−1^). Particle-size metrics included Mass Median Aerodynamic Diameter (MMAD), Geometric Standard Deviation (GSD), and the 16th (D16) and 84th (D84) percentile aerodynamic diameters. Fine particle metrics included Fine Particle Fraction (FPF, %), defined as the aerosol mass below 5 µm aerodynamic diameter recovered downstream of the NHF pathway, expressed as a percentage of the total aerosol mass recovered downstream of the NHF circuit. Fine Particle Dose (FPD, µg) was defined as the corresponding mass of respirable aerosol recovered downstream of the NHF pathway. Delivery-efficiency metrics included Delivered Fraction (DF), defined as the proportion of the loaded dose recovered downstream of the NHF pathway. Detailed definitions, calculation procedures, APSD interpolation methods, and numerator-denominator relationships for all aerosol-performance endpoints are provided in Supplementary Material S2. Each treatment combination was performed in triplicate, yielding 48 experimental runs. The full factorial design, randomization, run order, factors, and responses are provided in Supplementary Material S3.

### 2.5. Nebulisation Protocol

For each experimental run, 2.5 mL of formulation was nebulized to dryness. Nebulization was initiated only after the system stabilization period had elapsed. Nebulization was considered complete when no further visible aerosol was observed at the nebulizer outlet at the humidification chamber. Nebulization time ranged between approximately 6 and 9 min. No breathing simulation was used; all generated aerosol was continuously entrained and drawn through the NHF circuit towards the NGI. After each run, NGI collection cups were visually inspected. Runs were rejected and repeated if liquid water had completely evaporated leaving crystalline residue or if droplet deposits were distorted in NGI stages. The number, experimental-condition distribution, reasons for rejection, disposition of rejected and repeated runs, and timing of the exclusion criteria are reported in Supplementary Material S3 and Table S3b. Salbutamol sulfate deposited was recovered from the various components by rinsing surfaces with a known volume of distilled water and quantified using an ultraviolet spectrophotometric assay (Specord 250 plus, Analytik Jena GmbH, Jena, Germany) at 276 nm. Calibration curves were generated from serial dilutions of the study formulations over a concentration range of 0.78–200 µg·mL^−1^ for ISS and 0.39–100 µg·mL^−1^ for 3% HSS. Distilled water, sterile isotonic saline (0.9%), and hypertonic saline (3%) blanks exhibited zero absorbance at 276 nm. Additional details regarding calibration procedures are provided in Supplementary Material S4.

### 2.6. Regional Deposition

For regional deposition analysis, the deposited drug across the experimental setup was structured into four physiologically and experimentally interpretable partitions: pre-device drug loss, NHF circuit deposition, nasal cavity deposition, and NGI deposition (Figure 1). Pre-device drug loss was defined as drug recovered from the ampoule prior to entry into the experimental setup. NHF circuit deposition was defined as the cumulative fraction of recovered dose retained upstream of the nasal airway model. This region comprised the nebulizer, humidification chamber, heated breathing tube, and cannula. Nasal cavity deposition was defined as the fraction of recovered dose retained within the nasal airway model. NGI deposition was defined as the cumulative fraction of recovered dose on NGI stages S1–S7 and the micro-orifice collector (MOC) and filter. Regional deposition therefore refers to the salbutamol mass recovered from each defined experimental region. The values were not corrected for potential differences in recovery efficiency among component materials or surface geometries. Further details of the analytical procedure and its recovery-related limitations are provided in Supplementary Material S4.

### 2.7. Mass Balance and Recovery

Total salbutamol recovery was calculated as the sum of drug recovered from all experimental components. Across accepted experimental runs, total recovery ranged from 90.4% to 102.4% of the nominal loaded dose for the ISS formulation and from 90.3% to 100.7% for the 3% HSS formulation, confirming acceptable mass balance throughout the study. Recovery values for individual experimental runs are provided in Supplementary S5.

### 2.8. Statistical Analyses

All statistical analyses were performed in R version 4.4.1 (R Foundation for Statistical Computing, Vienna, Austria) using prespecified scripts developed for this study. Aerosol characteristic endpoints were analyzed using a balanced factorial framework with NGI/system temperature, formulation, and NHF rate treated as fixed factors. The primary model (Response ~ Temperature × Formulation × Flow) was used to estimate main effects and interaction terms.

To assess within-formulation behavior, separate formulation-specific models (Response ~ Temperature × Flow) were fitted for the ISS formulation and the 3% HSS formulation. Matched isotonic versus hypertonic contrasts were performed at each temperature–flow combination using Welch two sample *t* tests. Pairwise Welch comparisons were prespecified and used to support interpretation of factorial effects. To assess the robustness of exploratory pairwise comparisons, a Benjamini–Hochberg false discovery rate (FDR) sensitivity analysis was performed. The primary statistical inference remained based on the factorial ANOVA framework.

Between-formulation comparisons of FPD were performed on a dose-normalized basis to account for differing nominal loaded doses. Bounded percentage outcomes were analyzed on the logit scale for inference, whereas descriptive statistics are reported on the original scale. Factorial results are summarized as type III ANOVA outputs with associated *p* values and partial eta squared effect sizes for selected endpoints.

Stage-wise deposition data were further analyzed using groupings (NHF circuit deposition, nasal cavity deposition, and NGI deposition) to support mechanistic interpretation of deposition behavior. Pre-device drug loss was excluded from the regional deposition models. All summary data are presented as mean ± standard deviation (SD), and statistical significance was interpreted at *p* < 0.05. Full statistical details, including contrast coding, model diagnostics, full factorial results, and FDR sensitivity analyses, are provided in Supplementary Material S6–S10.

## 3. Results

### 3.1. Particle-Size Metrics

Particle-size metrics consisting of MMAD, D84, D16, and GSD were analyzed using a balanced factorial framework, with NGI/system temperature, formulation, and flow treated as fixed factors.

#### 3.1.1. Particle-Size Metrics: ISS Formulation

Within the ISS formulation, particle-size behavior was strongly influenced by both NGI/System temperature and NHF, with MMAD showing highly significant main effects of temperature and flow, but no significant temperature × flow interaction. The statistical analysis summary is presented in Table 1. Similar to aerodynamic size, D84 was sensitive to temperature and flow but not to their interaction. D16 showed a similar trend with significant temperature and flow effects, along with a significant temp × flow interaction effect. GSD exhibited a weaker temperature effect, no independent flow effect, but a strong interaction, indicating that distribution breadth changed in a condition-specific rather than monotonic manner.

The descriptive data showed a clear decrease in the size of the ISS aerosol with increased NGI/system temperature and higher NHF. Temperature- and flow-dependent particle-size responses for the ISS formulation are shown in Figure 2. MMAD decreased from 2.36 ± 0.09 µm at 5 °C/30 L·min^−1^ to 1.44 ± 0.17 µm at 5 °C/50 L·min^−1^, and further to 1.16 ± 0.05 µm at 40 °C/ 50 L·min^−1^. A similar directional response was observed for D16, which decreased from 1.36 ± 0.03 µm at 5 °C/30 L·min^−1^ to 0.63 ± 0.02 µm at 40 °C/50 L·min^−1^. D84 also declined across the same range, from 3.87 ± 0.08 µm to 1.79 ± 0.20 µm, indicating that both the central and upper portions of the ISS particle-size distribution shifted downward as NGI/system temperature and flow conditions intensified.

GSD behaved differently from the other size metrics. Although the ISS aerosol remained relatively narrow overall, the breadth of the distribution was not constant across conditions. GSD was greatest at 5 °C/50 L·min^−1^ (1.90 ± 0.04) and lowest at 40 °C/50 L·min^−1^ (1.69 ± 0.07), with intermediate values at 21 °C and 37 °C. This pattern is consistent with the significant temperature × flow interaction and indicates that, under the ISS condition, shifts in the upper and lower percentiles of the distribution were not always proportional across the full temperature and flow matrix.

These data indicate that the ISS formulation exhibited progressively smaller measured aerodynamic particle-size as NHF-conditioned transport intensified, particularly under higher flow and NGI/system temperature. The sensitivity of MMAD, D84 and D16 to flow, combined with the interaction observed for D16 and GSD, suggests that ISS aerosol size under NHF is not determined by a single thermal or flow effect in isolation, but by how these conditions jointly reshape the transported distribution.

#### 3.1.2. Particle-Size Metrics: 3% HSS Formulation

The 3% HSS formulation demonstrated even stronger thermal sensitivity than the ISS formulation. The statistical analysis summary is presented in Table 2. MMAD was strongly affected by both temperature and flow and the temperature × flow interaction. D84 and D16 were also strongly responsive to both temperature and flow, but their interaction terms were not significant. GSD again showed a smaller but significant temperature effect, with no independent flow effect and no evidence of interaction.

The descriptive statistics revealed a marked narrowing of hypertonic particle size under higher NGI/system temperatures and flow conditions. Temperature- and flow-dependent particle size responses for the 3% HSS formulation are shown in Figure 3. MMAD decreased from 2.78 ± 0.01 µm at 5 °C/30 L·min^−1^ to 2.28 ± 0.04 µm at 5 °C/50 L·min^−1^, then progressively to 1.30 ± 0.04 µm at 40 °C/50 L·min^−1^. D84 likewise declined from 4.26 ± 0.05 µm to 2.33 ± 0.20 µm. D16 showed the same directional contraction, decreasing from 1.51 ± 0.01 µm at 5 °C/30 L·min^−1^ to 0.67 ± 0.02 µm at 40 °C/50 L·min^−1^, confirming that the overall hypertonic size distribution shifted to substantially smaller aerodynamic sizes across the studied NGI/system temperature range.

Compared with the ISS formulation, the 3% HSS aerosol showed a more pronounced thermodynamic dependence of MMAD and a clearer temperature × flow coupling. This was particularly evident in the steep reduction in MMAD between 21 °C and 40 °C under both airflow settings, and in the large effect sizes for temperature and flow. In contrast, GSD remained within a relatively narrow range (1.68–1.87) despite the substantial changes in MMAD, D16 and D84, indicating that the dominant effect of NGI/System temperature in the 3% HSS formulation was a translational shift in the distribution toward smaller sizes rather than a major restructuring of polydispersity. Particle-size metrics for the 3% HSS formulation are shown in Figure 3.

#### 3.1.3. Particle-Size Metrics: Isotonic Versus Hypertonic Comparison

Matched isotonic–hypertonic comparisons demonstrated that the impact of formulation on particle size was highly condition-dependent. For MMAD, the 3% HSS formulation was significantly larger than the ISS formulation at 5 °C/30 L·min^−1^ (difference = −0.419 µm, 95% CI −0.639 to −0.199, *p* = 0.014) and 5 °C/50 L·min^−1^ (difference = −0.841 µm, 95% CI −1.244 to −0.438, *p* = 0.0109). Significant differences in the same direction were also observed at 21 °C/30 L·min^−1^ (difference = −0.457 µm, *p* = 0.0327) and 21 °C/50 L·min^−1^ (difference = −0.285 µm, *p* = 0.00139). At 37 °C, MMAD no longer differed significantly between formulations at either flow setting, indicating substantial convergence. At 40 °C/30 L·min^−1^, the direction of the difference reversed, with ISS MMAD becoming significantly larger than 3% HSS MMAD (difference = +0.207 µm, 95% CI 0.088 to 0.326, *p* = 0.0115), whereas at 40 °C/50 L·min^−1^ the 3% HSS formulation remained only modestly larger (difference = −0.144 µm, *p* = 0.0246). These matched differences across temperature–flow conditions are summarized in Figure 4.

D84 and D16 showed the same broad pattern. Under colder and intermediate NGI/System temperature conditions, the 3% HSS formulation generally retained larger percentile diameters, especially at 5 °C/50 L·min^−1^ where the isotonic–hypertonic differences were −0.564 µm for D16 (*p* = 2.37 × 10^−4^) and −1.127 µm for D84 (*p* = 6.23 × 10^−4^). At warmer conditions, particularly 40 °C/30 L·min^−1^, these differences again converged and, in some cases, reversed, with ISS D16 (+0.155 µm, *p* = 0.00839) and D84 (+0.289 µm, *p* = 0.00915) exceeding the corresponding 3% HSS values. Thus, the direction and magnitude of formulation-dependent size differences were not fixed but varied with the NGI/System temperature and flow environment.

GSD showed smaller and less consistent between-formulation differences than the size metrics themselves. At several conditions, the ISS formulation exhibited a broader distribution than the 3% HSS formulation, most notably at 5 °C/50 L·min^−1^, whereas at other conditions the 3% HSS formulation showed slightly broader distributions. Several contrasts were non-significant, supporting the view that formulation primarily altered overall particle size rather than producing a uniformly different width of the distribution.

Taken together, the matched contrasts indicate that the 3% HSS formulation entered the cooler NGI/System with a consistently larger aerodynamic size profile than the ISS formulation, but that these differences diminished, disappeared, or even inverted under warmer NGI/System conditions. In practical terms, formulation identity alone did not determine final particle size. The observed aerosol behavior depended on the coupled interaction between formulation composition, NHF, and the NGI/System thermal environment. This interpretation is consistent with the within-formulation models, in which MMAD showed a significant temperature × flow interaction for the 3% HSS formulation but only a borderline interaction for the ISS formulation.

### 3.2. Fine Particle Metrics

Fine particle metrics were examined to determine whether the changes in aerosol size were translated into differences in respirable drug output. FPF (%) describes the respirable proportion of the transported aerosol, whereas dose-normalized FPD (%) quantifies the corresponding fine particle mass relative to the nominal loaded dose.

#### 3.2.1. Fine Particle Metrics: ISS Formulation

Within the ISS formulation, the FPF (%) was significantly affected by downstream temperature (*p* = 0.0174) and NHF (*p* < 0.0001), and temperature × flow interaction (*p* = 0.0002). The dose-normalized FPD showed the same overall pattern, with significant effects of temperature (*p* = 0.0005), flow (*p* = 0.0006), and interaction (*p* = 0.0001). These interactions indicate that within the ISS formulation, fine-particle behavior was jointly shaped by NGI/system temperature and NHF. Temperature-dependent FPF and FPD responses for the ISS formulation are shown in Figure 5.

Descriptive data showed that fine particle metrics did not follow the same pattern as the particle-size descriptors. Although MMAD, D16, and D84 decreased progressively with increasing temperature and flow (Section 3.1), corresponding changes in FPF (%) were not monotonic. At 30 L·min^−1^, FPF increased from 83.8 ± 1.2% at 5 °C to 93.6 ± 1.6% at 37 °C before decreasing to 88.1 ± 2.0% at 40 °C. At 50 L·min^−1^, FPF remained high across all conditions but declined from approximately 99% at 5–21 °C to approximately 94–95% at 37–40 °C. Thus, reductions in aerodynamic particle size were not consistently accompanied by increases in respirable fraction (Figure 5).

A different pattern was observed for FPD. At 30 L·min^−1^, FPD increased from 2586 ± 48 µg at 5 °C to 2869 ± 96 µg at 37 °C, before decreasing to 2682 ± 190 µg at 40 °C. At 50 L·min^−1^, FPD increased from 2661 ± 212 µg at 5 °C to 2953 ± 146 µg at 21 °C, then decreased to 2115 ± 87 µg at 37 °C and 2060 ± 211 µg at 40 °C, reflecting the decreased delivery efficiency of the NHF pathway under these higher temperature conditions (see Section 3.3). As a result, the conditions associated with the smallest MMAD values did not correspond to the highest FPD values (Figure 5).

A comparison between NHF rate conditions further demonstrated that higher respirable fraction did not necessarily correspond to higher respirable dose. Across all downstream temperatures, FPF was consistently higher at 50 L·min^−1^ than at 30 L·min^−1^, remaining approximately 94–99% at 50 L·min^−1^ compared with 84–94% at 30 L·min^−1^. Despite this, FPD was generally higher at 30 L·min^−1^ across the temperature range. For example, at 37 °C, FPF was 94.9% at 50 L·min^−1^ compared with 93.6% at 30 L·min^−1^, yet FPD was substantially lower at 50 L·min^−1^ (2115 µg vs. 2869 µg). Similar behavior was observed at 40 °C, where FPF remained higher at 50 L·min^−1^ (94.8% vs. 88.1%), while FPD remained lower (2060 µg vs. 2682 µg) (Figure 5).

#### 3.2.2. Fine Particle Metrics: 3% HSS Formulation

Within the 3% HSS formulation, the FPF (%) was strongly affected by NGI/System temperature (*p* < 0.0001) and NHF (*p* < 0.0001), whereas the temperature × flow interaction was not significant (*p* = 0.2182). In contrast, the dose-normalized FPD showed significant effects of temperature (*p* < 0.0001), flow (*p* < 0.0001), and interaction (*p* = 0.0012). Thus, within the 3% HSS formulation, the respirable fraction itself varied primarily with the independent effects of temperature and flow, whereas the corresponding fine-particle mass remained sensitive to the joint downstream thermal–flow environment. Temperature-dependent FPF and FPD responses for the 3% HSS formulation are shown in Figure 6.

Descriptive data showed that reductions in MMAD, D16, and D84 with increasing temperature were generally accompanied by increases in FPF (%). At 30 L·min^−1^, FPF increased from 73.76 ± 2.00% at 5 °C to 95.99 ± 2.00% at 40 °C. Similarly, at 50 L·min^−1^, FPF increased from 92.95 ± 3.94% at 5 °C to 98.09 ± 0.26% at 40 °C. However, the magnitude of the FPF response differed between flow conditions. At 30 L·min^−1^, an approximately 22% increase in FPF was observed between 5 °C and 40 °C, whereas at 50 L·min^−1^ the corresponding increase was approximately 5% despite a similarly large reduction in MMAD (Section 3.1).

A different pattern was observed for FPD. At 30 L·min^−1^, FPD increased progressively from 855 ± 2 µg at 5 °C to 1191 ± 2 µg at 37 °C and 1202 ± 28 µg at 40 °C. In contrast, at 50 L·min^−1^, FPD varied only modestly, increasing from 779 ± 69 µg at 5 °C to 829 ± 36 µg at 40 °C despite substantial reductions in MMAD and consistently high FPF values (Figure 6). As a result, the conditions associated with the largest reductions in aerodynamic particle size did not necessarily correspond to the largest increase in FPD.

Comparison between NHF rate conditions revealed a pattern similar to that observed for the ISS formulation, with FPF consistently higher at 50 L·min^−1^ than at 30 L·min^−1^ across all temperature conditions. For example, at 5 °C, FPF was 92.95% at 50 L·min^−1^ compared with 73.76% at 30 L·min^−1^, and at 40 °C, FPF was 98.09% compared with 95.99%. Despite these higher respirable fractions, FPD remained lower at 50 L·min^−1^ than at 30 L·min^−1^ throughout the experimental matrix (Figure 6). At 40 °C, FPD was 829 µg at 50 L·min^−1^ compared with 1202 µg at 30 L·min^−1^, while at 37 °C the corresponding values were 902 µg and 1191 µg, respectively. Thus, although a greater proportion of recovered aerosol was respirable at the higher NHF rate, less respirable aerosol mass was delivered relative to the nominal loaded dose.

#### 3.2.3. Fine Particle Metrics: Isotonic Versus Hypertonic Comparison

Matched isotonic–hypertonic comparisons demonstrated condition-dependent differences in fine-particle metrics. At 5 and 21 °C NGI/System temperatures, the ISS formulation produced higher FPF at 5 °C/30 L·min^−1^ (difference = +10.05 percentage points, *p* = 0.0036). Conversely, at 37 and 40 °C NGI/system temperatures the direction reversed at 37 °C/50 L·min^−1^ (difference = −2.60 percentage points, *p* = 0.0265) and 40 °C/30 L·min^−1^ (difference = −7.92 percentage points, *p* = 0.0084), indicating higher FPF for the 3% HSS formulation at those settings. No significant formulation difference in FPF was detected at 5 °C/50 L·min^−1^, 21 °C/30 L·min^−1^, 37 °C/30 L·min^−1^, or 40 °C/50 L·min^−1^. Although the contrast at 21 °C/50 L·min^−1^ was nominally significant before adjustment (difference = +8.23 percentage points, raw *p* = 0.0451), it did not remain significant following FDR correction (q = 0.0657).

Because the two formulations differed in nominal loaded dose, formulation comparisons for FPD were based on dose-normalized FPD (%). In contrast to FPF, dose-normalized FPD (%) showed a more consistent ISS advantage across the experimental matrix. Significant ISS advantages were observed at 5 °C/30 L·min^−1^ (+17.54 percentage points, *p* = 0.0009), 21 °C/30 L·min^−1^ (+14.83 percentage points, *p* = 0.007), 5 °C/50 L·min^−1^ (+22.06 percentage points, *p* = 0.003), and 21 °C/50 L·min^−1^ (+26.3 percentage points, *p* = 0.0007). Although the magnitude of the difference narrowed at higher NGI/System temperatures, the ISS formulation remained numerically higher across all temperature–flow combinations. Significant ISS-favoring differences remained evident at 37 °C/30 L·min^−1^ (+9.73 percentage points, *p* = 0.0127) and 37 °C/50 L·min^−1^ (+6.23 percentage points, *p* = 0.0197). At 40 °C, the differences were smaller and did not reach statistical significance, reflecting partial convergence of dose-normalized FPD between formulations. The differing formulation response patterns for FPF and dose-normalized FPD are summarized in Figure 7.

### 3.3. NHF Delivery Efficiency

DF was used as a system-level measure of NHF delivery efficiency, representing the proportion of the loaded dose that was recovered after transport through the complete NHF pathway.

#### 3.3.1. NHF Delivery Efficiency: ISS Formulation

Within the ISS formulation, DF was significantly affected by NHF rate (*p* = 0.0002), whereas the effects of downstream temperature (*p* = 0.0649) and the temperature × flow interaction (*p* = 0.0982) did not reach statistical significance. Thus, delivery efficiency within the ISS formulation was primarily governed by NHF rate rather than by NGI/system temperatures.

Descriptively, DF remained relatively stable at 30 L·min^−1^, varying between 61.60 ± 2.61% and 62.37 ± 1.20% across the temperature range. At 50 L·min^−1^, DF remained relatively high at 21 °C (59.77 ± 3.03%) but decreased markedly at 37 °C (44.76 ± 2.05%) and remained low at 40 °C (43.98 ± 5.35%). This reduction was reflected in the dose-normalized FPD results reported above, which also declined at 37 °C and 40 °C (Figure 8).

#### 3.3.2. NHF Delivery Efficiency: 3% HSS Formulation

Within the 3% HSS formulation, DF was significantly affected by both NGI/system temperature and NHF rate. Flow was the dominant factor, with a very large effect (*p* < 0.0001), while temperature also showed a significant effect (*p* = 0.0032). The temperature × flow interaction was not significant (*p* = 0.3642), indicating that the effects of temperature and flow were largely additive rather than interaction-dependent (Figure 8). At 30 L·min^−1^, DF increased from 46.37 ± 1.27% at 5 °C to approximately 50% under all higher temperature conditions. At 50 L·min^−1^, DF remained substantially lower, ranging from 33.48 ± 1.63% to 36.98 ± 0.84%.

#### 3.3.3. NHF Delivery Efficiency: Isotonic Versus Hypertonic Comparison

Matched formulation comparisons showed that DF was generally higher for the ISS formulation than for the 3% HSS formulation across the temperature–flow matrix. Significant ISS-favoring differences were observed at 5 °C/30 L·min^−1^ (*p* < 0.0001), 5 °C/50 L·min^−1^ (*p* = 0.012), 21 °C/50 L·min^−1^ (*p* = 0.001), 37 °C/30 L·min^−1^ (*p* = 0.006), and 37 °C/50 L·min^−1^ (*p* = 0.013). Although the magnitude of the formulation difference narrowed under some warmer conditions, DF remained numerically higher for the ISS formulation at all matched temperature–flow combinations.

The largest formulation differences were observed at the higher NHF rate and cooler-to-intermediate NGI/System temperatures. At 5 °C/50 L·min^−1^ and 21 °C/50 L·min^−1^, DF for the ISS formulation exceeded that of the 3% HSS formulation by approximately 20.9 and 23.8 percentage points, respectively. Although these differences narrowed at higher NGI/System temperatures, DF remained numerically higher for the ISS formulation across all matched temperature–flow combinations.

In addition to the observations described above, full-factorial analysis demonstrated significant Temperature × Formulation × Flow interactions across both particle-size and fine-particle metrics. Significant three-way interactions were observed for MMAD (*p* = 0.0055), D16 (*p* < 0.0001), GSD (*p* = 0.0009), FPF (*p* = 0.0042), and dose-normalized FPD (*p* = 0.0149). By contrast, no significant three-way interaction was observed for DF (*p* = 0.1342). These findings indicate that the influence of NGI/system temperature depended simultaneously on both formulation and NHF, with the strongest three-way interaction observed for D16.

### 3.4. Regional Deposition Profiles and Dose Redistribution

Regional deposition was analyzed on the % recovered mass basis to determine how recovered salbutamol mass was redistributed across the complete NHF aerosol pathway and is summarized in Figure 9. To facilitate interpretation of absolute aerosol delivery, corresponding regional deposition data expressed as a percentage of nominal loaded dose are provided in Supplementary Material S11.

#### 3.4.1. Pre-Device Drug Loss

Pre-device drug loss was small for the ISS formulation, ranging between approximately 0.3–1.4% (Supplementary Material S12), with no clear monotonic dependence on temperature or flow. Pre-device drug loss was zero by design for the 3% HSS formulation because the formulation was transferred directly into the nebulizer. Consequently, no between-formulation comparisons were performed.

#### 3.4.2. Nasal-Cavity Deposition

For the ISS formulation, nasal-cavity deposition ranged from 0.14 ± 0.12% to 3.29 ± 1.03% recovered mass. For the 3% HSS formulation, values ranged from 0.20 ± 0.06% to 3.53 ± 0.06%. Most conditions exhibited nasal deposition below 1% recovered mass, with only isolated peaks observed at 5 °C/30 L·min^−1^ for the 3% HSS formulation (3.53 ± 0.06%) and at 40 °C/30 L·min^−1^ for the ISS formulation (3.29 ± 1.03%).

Therefore, although statistically detectable variation was present, nasal-cavity deposition contributed only a small proportion of the recovered salbutamol mass compared with NHF circuit deposition (33–65%) and NGI deposition (34–65%). The major regional redistribution patterns observed in Figure 9 were therefore driven predominantly by shifts between the NHF circuit and NGI compartments rather than by changes in nasal-cavity deposition.

#### 3.4.3. NHF Circuit Deposition

NHF circuit deposition represented recovered salbutamol retained within the delivery pathway before the nasal cavity and NGI regions. The NHF circuit deposition was significantly affected by both temperature and flow for both formulations. For ISS, temperature was significant (*p* = 0.0193) and flow was strongly significant (*p* < 0.0001), while the temperature × flow interaction approached but did not reach the conventional significance threshold (*p* = 0.0563). For 3% HSS formulation, temperature was significant (*p* = 0.00047) and flow showed a very large effect (*p* < 0.0001), whereas the interaction was not significant (*p* = 0.3052). Figure 9 shows that NHF circuit deposition occupied a substantially larger proportion of recovered mass for the 3% HSS formulation, particularly at 50 L·min^−1^.

The 3% HSS formulation exhibited consistently high circuit retention, especially at 50 L·min^−1^. At 5 °C, NHF circuit deposition increased from 51.85 ± 1.33% at 30 L·min^−1^ to 64.96 ± 1.23% at 50 L·min^−1^. The same flow-related increase was present at each temperature: 46.15 ± 0.60% to 60.58 ± 2.85% at 21 °C, 47.01 ± 2.05% to 60.09 ± 0.35% at 37 °C, and 46.56 ± 1.86% to 63.16 ± 2.34% at 40 °C. Thus, approximately 60–65% of the recovered 3% HSS aerosol remained within the NHF pathway at the higher flow condition.

The ISS formulation showed lower circuit deposition overall and a less device-dominated distribution. At 30 L·min^−1^, ISS NHF circuit deposition was relatively stable across temperature, ranging from 33.27 ± 2.06% at 5 °C to 35.19 ± 6.45% at 40 °C. In contrast, at 50 L·min^−1^, NHF circuit deposition increased from 39.24 ± 2.03% at 21 °C to 51.89 ± 2.74% at 37 °C and 53.93 ± 6.48% at 40 °C. Thus, ISS circuit retention also increased at higher flow, but the magnitude of circuit dominance was generally smaller than that for the 3% HSS formulation. This temperature increase in circuit retention at 50 L·min^−1^ coincided with the reductions in dose-normalized FPD and DF reported earlier.

Matched formulation contrasts confirmed greater circuit retention for the 3% HSS formulation. Significant 3% HSS-favoring differences were observed at 5 °C/30 L·min^−1^ (−18.6%, *p* = 0.001), 5 °C/50 L·min^−1^ (−22.7%, *p* = 0.002), 21 °C/50 L·min^−1^ (−21.3%, *p* = 0.004), 37 °C/30 L·min^−1^ (−12.8%, *p* = 0.001), and 37 °C/50 L·min^−1^ (−8.2%, *p* = 0.001), where negative values indicate greater NHF circuit deposition for the 3% HSS formulation. At 21 °C/30 L·min^−1^, 40 °C/30 L·min^−1^, and 40 °C/50 L·min^−1^, the same 3% HSS-favoring direction was present numerically, but the matched differences did not reach statistical significance (*p* > 0.05).

Collectively, NHF circuit deposition was one of the clearest regional endpoints differentiating formulation behavior. Figure 9 shows that the 3% HSS formulation consistently partitioned a greater proportion of recovered aerosol into the NHF circuit compartment, particularly at 50 L·min^−1^, whereas the ISS formulation retained a larger proportion of recovered mass within the downstream NGI compartment.

#### 3.4.4. NGI Deposition

NGI deposition was defined as the total recovered mass (%) in NGI stages S1–S7 together with the combined MOC/filter collection stage. For ISS, NGI deposition was significantly affected by temperature (*p* = 0.0011), flow (*p* < 0.0001), and the temperature × flow interaction (*p* = 0.0230). At 30 L·min^−1^, NGI deposition remained relatively stable across the temperature range, varying between 60.15 ± 5.24% and 65.41 ± 7.82%. At 50 L·min^−1^, however, a marked redistribution was evident. NGI deposition remained high at 5 °C and 21 °C (56.53 ± 1.50% and 60.18 ± 1.91%, respectively) but declined to 45.41 ± 2.54% at 37 °C and 43.12 ± 5.27% at 40 °C (Figure 9). This indicates that ISS generally retained a more downstream, NGI-oriented distribution, although warmer high-flow conditions shifted the balance toward greater circuit retention.

For the 3% HSS formulation, NGI deposition was also significantly affected by temperature (*p* < 0.0001), flow (*p* < 0.0001), and the temperature × flow interaction (*p* = 0.0483). At 30 L·min^−1^, NGI deposition increased from 44.62 ± 1.35% at 5 °C to approximately 53% at 21–40 °C. This increase corresponded with the reduction in NHF circuit retention observed over the same temperature range (Figure 9). At 50 L·min^−1^, NGI deposition remained substantially lower, varying between 33.86 ± 1.88% and 39.40 ± 0.56%, reflecting the persistently high circuit retention observed under these conditions.

These findings show that NGI deposition was strongly flow-dependent and that the temperature effect varied by flow, particularly for the ISS formulation. These significant interactions are shown in the NGI deposition panels of Figure 9. The significant T × F interaction for NGI deposition did not necessarily reflect a crossover between flow conditions. Instead, as shown in Figure 9, NGI deposition was generally lower at 50 L·min^−1^ than at 30 L·min^−1^, but the magnitude of this flow-related reduction varied across temperatures. Thus, the interaction reflects a temperature-dependent change in the size of the flow effect rather than a reversal of the flow effect.

Matched contrasts illustrated this formulation difference most clearly at 50 L·min^−1^. At 5 °C/50 L·min^−1^, NGI deposition was 56.53 ± 1.50% for the ISS formulation compared with 33.86 ± 1.88% for the 3% HSS formulation. At 21 °C/50 L·min^−1^, the corresponding values were 60.18 ± 1.91% and 36.80 ± 3.50%, respectively. At higher NGI/System temperatures, these differences narrowed at 37 °C (45.41 ± 2.54% versus 39.40 ± 0.56%) and at 40 °C (43.12 ± 5.27% versus 36.55 ± 2.22%). These high-flow data show that the 3% HSS formulation consistently produced less NGI deposition than the ISS formulation, with the largest separations occurring at 5 °C and 21 °C.

At 30 L·min^−1^, the two formulations were closer at warmer temperatures, but the overall pattern still favored higher NGI deposition for ISS. The most pronounced 30 L·min^−1^ separation occurred at 5 °C, where ISS NGI deposition was 64.60 ± 2.83% compared with 44.62 ± 1.35% for 3% HSS. At 21 °C, 37 °C, and 40 °C, 3% HSS NGI deposition increased to approximately 52–54%, whereas ISS remained around 60–65%, narrowing but not eliminating the formulation difference.

Overall, Figure 9 demonstrates that the principal effect of formulation, temperature, and flow was redistribution of recovered aerosol mass between the NHF circuit and NGI compartments. Conditions associated with increased NHF circuit retention exhibited reduced NGI deposition, whereas conditions associated with reduced circuit retention exhibited a more NGI-dominant recovered-mass distribution.

## 4. Discussion

### 4.1. Interaction of NHF, NGI/System Temperature, and Formulation

The principal finding of this study was that aerosol characteristics during NHF delivery were determined by a hierarchy of interacting factors, with NHF rate exerting the greatest influence on downstream delivery. The effect of NGI/system temperature was strongly formulation dependent and became most apparent when considered in combination with NHF. Across almost all delivery and deposition-related endpoints, increasing NHF rate from 30 to 50 L·min^−1^ reduced downstream aerosol delivery. This was reflected by lower DF, lower FPD, and reduced NGI deposition accompanied by increased NHF circuit retention. The influence of NGI/system temperature emerged primarily through its interaction with formulation composition and became most evident when considered in conjunction with NHF. This interaction structure was further supported by significant Temperature × Formulation × Flow interactions observed for MMAD, D16, GSD, FPF, and dose-normalized FPD, demonstrating that the effects of NGI/System temperature conditions could not be interpreted independently. Collectively, these findings indicate that aerosol characteristics following NHF-conditioned transport could not be explained by flow, formulation, or temperature independently but by the combined interaction of all three factors.

The matched isotonic-versus-hypertonic comparisons provide the clearest evidence of this interaction. Under lower NGI/system temperatures, particularly at 50 L·min^−1^, the 3% HSS formulation exhibited larger MMAD and D84 values, lower FPD, lower DF, greater NHF circuit deposition, and lower NGI deposition than the ISS formulation. As NGI/system temperatures increased, these formulation differences progressively narrowed across multiple endpoint classes. However, this convergence was neither complete nor uniform. Differences in aerodynamic particle size became progressively smaller, whereas differences in FPD, DF, and regional deposition frequently remained evident. Hence, convergence in particle-size descriptors did not result in equivalent convergence in downstream aerosol delivery.

Another important observation was that temperature-induced changes in aerodynamic particle size did not translate uniformly into downstream aerosol delivery outcomes. Figure 10 demonstrates that increasing NGI/system temperature produced markedly different responses across aerodynamic particle size, fine-particle, delivery, and regional deposition endpoints. While reductions in MMAD were observed under all conditions, the magnitude and direction of the corresponding changes in FPF, FPD, DF, and NGI deposition varied considerably across formulations and NHF conditions. The clearest evidence of this behavior was observed for the 3% HSS formulation. Almost identical reductions in MMAD at 30 and 50 L·min^−1^ were associated with markedly different responses in FPF, FPD, DF, and NGI deposition (Figure 10). One possible explanation for this behavior is that the factors governing aerosol size distribution were not identical to those governing aerosol penetration through the NHF pathway. Reductions in MMAD would generally be expected to increase the proportion of aerosol contained within the respirable size range, consistent with the observed increases in FPF. However, the same transport conditions that influenced aerosol size also influenced aerosol losses within the delivery system. Thus, improvements in aerodynamic particle size were not always accompanied by equivalent improvements in FPD, DF, or NGI deposition. The regional deposition results support this interpretation, demonstrating that changes in aerosol transport and retention within the NHF pathway occurred concurrently with changes in APSD. Under these conditions, aerosol delivery depended not only on the aerodynamic characteristics of the aerosol itself but also on the efficiency with which aerosol mass survived transport through the complete NHF system. Collectively, these findings indicate that, under NHF conditions, the magnitude of change in the particle size did not consistently predict the magnitude of change in the downstream aerosol delivery.

Another notable feature of the dataset was that higher FPF did not necessarily produce higher respirable doses. In both formulations, 50 L·min^−1^ conditions frequently exhibited respirable fractions that were comparable to, or greater than, those observed at 30 L·min^−1^ yet produced lower absolute fine-particle doses. Thus, a greater proportion of respirable aerosol did not necessarily translate into a greater quantity of respirable aerosol reaching the impactor. This observation highlights an important distinction between aerosol size distribution and aerosol delivery efficiency. While FPF describes the proportion of aerosol contained within the respirable size range, FPD additionally reflects aerosol transport efficiency and aerosol survival throughout the NHF pathway. The regional deposition and DF results suggest that this discrepancy was driven, at least in part, by increased aerosol retention within the delivery system under higher-flow conditions. Accordingly, improvements in aerosol size distribution were not always accompanied by equivalent improvements in delivered respirable dose because gains in respirable fraction could be offset by increased transport losses before the aerosol reached the impactor. These findings demonstrate that respirable aerosol fraction and delivered respirable dose should be regarded as complementary rather than interchangeable indicators of aerosol characteristics during NHF.

The strong interaction between NHF rate, NGI/system temperature, and formulation observed in the present study highlights the importance of the physical environment in which aerosols are transported and subsequently characterized. This is particularly relevant under NHF conditions, where the aerosol reaching the NGI represents the aerodynamic state of the aerosol after transport through a heated and humidified pathway. Across the experimental matrix, the 3% HSS formulation consistently exhibited greater sensitivity to downstream thermal conditions than the ISS formulation, particularly for MMAD, D16, D84, FPF, and FPD. These findings are most consistent with formulation-dependent hygroscopic behavior and support hygroscopic growth as a plausible mechanistic explanation for the observed responses. Hygroscopic size changes are influenced by solute concentration, temperature, and humidity. Together, these factors affect the water-vapor concentration gradient between droplet surfaces and the surrounding environment [18,25,33]. More concentrated saline aerosols may exhibit a greater response to changes in thermal and humidity conditions than near-isotonic systems. Under NHF conditions, where aerosols are transported through a heated and humidified pathway, these formulation-dependent differences may influence the aerodynamic size distribution of aerosol ultimately presented to the impactor. In addition, the greater NHF circuit retention observed for the 3% HSS formulation suggests that larger 3% HSS droplets may have been preferentially removed during transport, progressively enriching the aerosol population reaching the impactor in smaller penetrating particles. This may help explain the convergence of ISS and 3% HSS APSD observed at higher NGI/system temperatures despite persistent differences in downstream aerosol recovery and regional deposition.

Collectively, these findings indicate that the ISS and 3% HSS formulations exhibited distinct aerosol characteristics measured following NHF-conditioned transport and differed in how those characteristics were translated into delivery and deposition outcomes. While previous studies have demonstrated that aerosol hygroscopicity influences particle-size evolution, the present findings extend this understanding by demonstrating that the ISS and 3% HSS formulations differed not only in APSD responses but also in how those responses were translated into delivered dose, aerosol recovery, and regional deposition outcomes under NHF conditions.

### 4.2. Implications for Testing and Standardization

Existing nebulizer and APSD testing standards were developed primarily for conventional interfaces. Under NHF conditions, however, the aerosol reaching the impactor reflects a combined effect of transport through the device, its components, and a nasal cannula [21]. The present findings demonstrate that NGI/System thermal conditions at which these aerosols are characterized can influence measured aerodynamic particle-size characteristics, supporting the view that NGI measurements closer to body temperature may provide a more representative assessment of the aerosol state present during NHF delivery.

Nevertheless, the practical significance of these temperature-dependent changes should be interpreted in the context of their absolute magnitude and the particle-size range in which they occurred. Although statistically significant reductions in MMAD and D84 were observed with increasing NGI/system temperature, most changes were less than 1 μm in magnitude, and aerosol size distributions remained within the conventionally defined respirable size fraction (≤5 µm aerodynamic diameter). MMAD values remained within approximately 1.1 to 2.9 µm and D84 values remained below approximately 5.3 µm under both conventional cooled conditions (5 °C) and chamber temperatures approaching body temperature (37 °C). Particles within the MMAD 1–3 µm range are governed predominantly by the same broad deposition processes, predominantly gravitational sedimentation, rather than by fundamentally different deposition mechanisms [34]. Therefore, although increasing NGI/system temperature altered measured APSD, these changes occurred largely within an already respirable aerosol population and would not be expected to produce major shifts in the dominant deposition mechanisms.

From a translational and methodological perspective, NGI measurements conducted at temperatures closer to body temperature provide mechanistic insight into aerosol evolution during transport through heated and humidified systems. This additional information may be particularly relevant for formulations exhibiting greater hygroscopic responsiveness, such as hypertonic aerosols, where formulation-dependent differences were expressed not only through MMAD but also through delivered dose, aerosol recovery, and regional deposition outcomes. Nevertheless, these findings suggest that conventional NGI methodologies remain a useful and practical basis for aerosol development, comparative characterization and device development under NHF conditions.

## 5. Limitations and Future Work

Several limitations should be considered when interpreting these findings. This study was performed using one NHF setup and one pair of isotonic–hypertonic formulations. Therefore, while the findings support a mechanistic role for formulation, flow, and thermal conditioning, direct generalization to all humidified aerosol systems, NHF interfaces, nebulizer configurations, and formulations should be made cautiously. The proposed role of formulation-dependent hygroscopic behavior was inferred from particle size, delivery, and regional-deposition outcomes. Droplet composition, density, hygroscopic growth, or aerosol equilibration during transport were not measured directly. In addition, the manipulated temperature factor was the downstream NGI/system environment rather than the humidifier temperature of the NHF device itself. Aerosol temperature, local humidity, and NGI component temperatures were not directly measured; therefore, the study compared controlled downstream thermal environments rather than directly measured aerosol thermal states.

The experimental setup also did not incorporate breathing simulation. Aerosol transport occurred under continuous entrainment conditions, and therefore the influence of patient-specific breathing patterns, airway residence times, and cyclic flow conditions on aerosol behavior was not assessed. Furthermore, the “NGI deposition” result represents an in vitro downstream-delivery surrogate under the present continuous-entrainment conditions and does not represent the exact dose that would reach a patient’s lungs. Similarly, the regional results were expressed as percentage of recovered mass; therefore, they describe redistribution of recovered salbutamol within the system and do not by themselves quantify the absolute aerosol mass surviving downstream (corresponding results expressed as percentages of nominal loaded dose are provided in Supplementary Material S11). In addition, component-specific salbutamol recovery experiments were not performed. Differences in recovery efficiency among the NHF circuit, nasal replica, and NGI could therefore have influenced the estimated regional-deposition proportions. Accordingly, the regional results represent the distribution of salbutamol recovered using the applied distilled-water rinsing and analytical procedures rather than recovery-corrected estimates of the absolute mass deposited in each region. Future work incorporating additional formulations, alternative NHF configurations, physiologically realistic breathing profiles, and in vivo or physiologically representative deposition measurements would help further define the clinical significance of the observed responses.

## 6. Conclusions

This study demonstrated that aerosol characteristics following NHF-conditioned transport are influenced by the combined interactions of NHF rate, formulation, and NGI/system temperature, with NHF exerting the greatest influence. The effects of these factors were interdependent. Aerosol characteristics under NHF conditions therefore could not be adequately explained by flow, formulation, or temperature alone, as reflected by significant three-way interactions across several particle metrics.

Although NGI/System thermal conditions produced significant changes in MMAD, D84, and D16, these changes did not translate uniformly into fine particle and delivery outcomes. Similar reductions in MMAD were frequently associated with markedly different responses in FPF, FPD, DF, and regional deposition. Likewise, higher respirable fractions did not necessarily result in greater respirable doses because aerosol transport losses within the NHF pathway remained an important determinant of downstream delivery. These findings demonstrate that aerodynamic particle size descriptors, fine-particle metrics, delivered dose, and regional deposition provide complementary—rather than interchangeable—measures of aerosol characteristics following NHF-conditioned transport.

The 3% HSS formulation exhibited substantially greater sensitivity to NGI/system thermal conditions than the ISS formulation across MMAD, FPF, FPD, aerosol recovery, and regional deposition endpoints. The present findings therefore extend current understanding by demonstrating that isotonic and hypertonic formulations differed not only in aerosol particle size evolution but also in how changes in aerosol state were translated into delivery and deposition outcomes during transport through a heated and humidified NHF system.

These findings indicate that measurements conducted at NGI/system temperatures approaching body temperature provide additional mechanistic insight into aerosol transformation during NHF delivery, particularly for hygroscopic formulations. However, because the observed APSD differences were relatively modest in absolute magnitude, and all aerosols remained within size ranges commonly associated with respirable aerosol, the results support the continued use of conventional NGI methodologies for routine aerosol development, formulation comparison, and performance characterization during NHF therapy. Taken together, aerosol characteristics governing downstream saline delivery during NHF are best understood in the context of the complete conditioned delivery pathway evaluated in the present study.

## Figures and Tables

**Figure 1:**
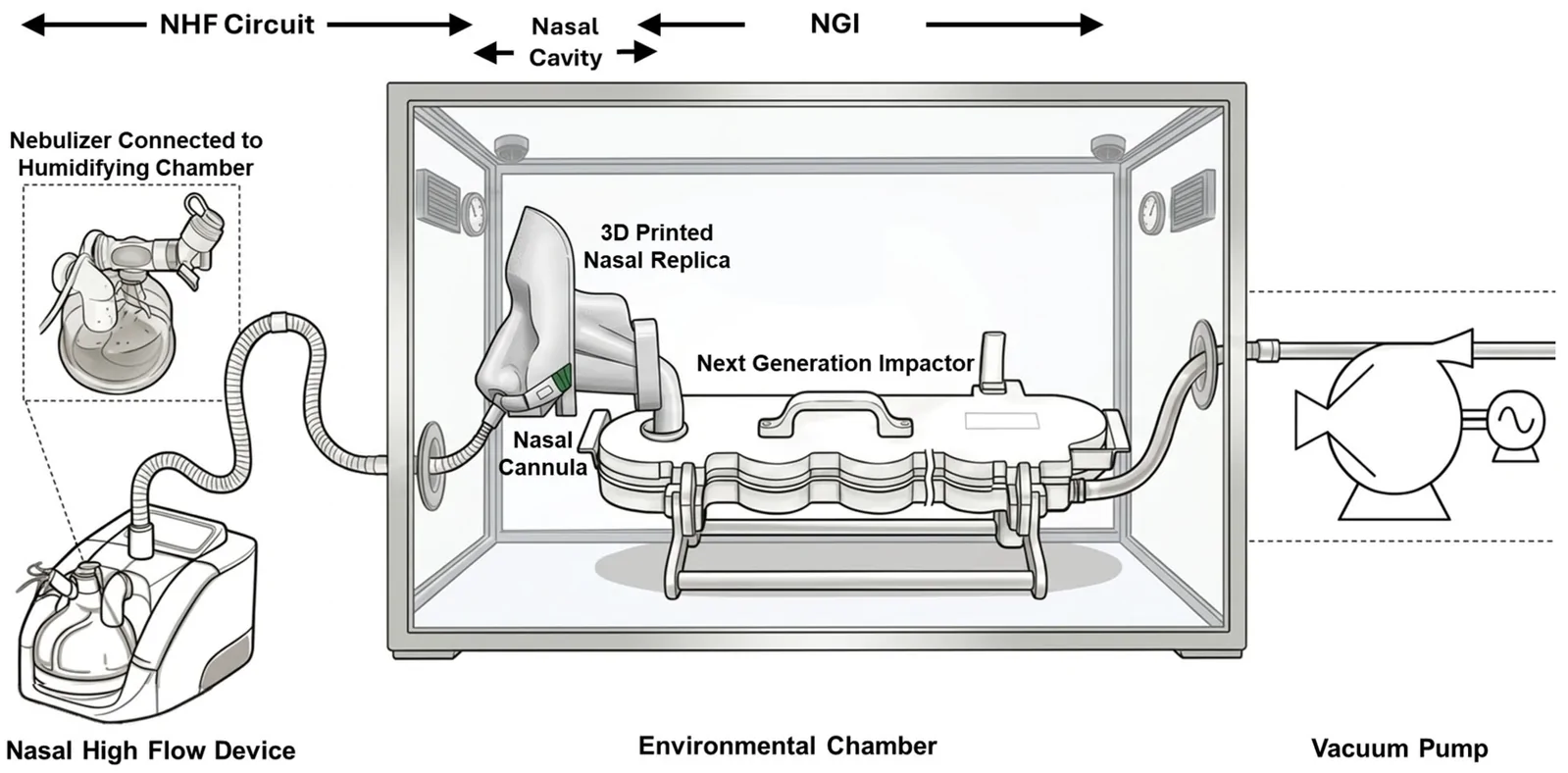
**Schematic of the experimental setup used to evaluate aerosol delivery under NHF-conditioned transport.** A vibrating mesh nebulizer was connected to the humidification chamber of the Airvo^TM^ 3 NHF device, with the device and nebulizer positioned outside the environmental chamber throughout all experiments. Aerosol generated upstream was transported through the heated breathing tube and nasal cannula into a 3D-printed adult nasal replica housed inside the environmental chamber. The nasal replica was coupled directly to a Next Generation Impactor (NGI), which was connected to a downstream vacuum pump. By enclosing the nasal replica and NGI within the environmental chamber, the downstream NHF-to-NGI pathway was exposed to controlled thermal conditions during aerosol transport and aerodynamic particle-size assessment. This configuration enabled evaluation of aerosol behavior after passage through a conditioned NHF pathway under defined downstream NGI/system temperature conditions. The figure also illustrates the partitioning used for regional deposition reporting: NHF circuit, nasal cavity, and NGI.

**Figure 2:**
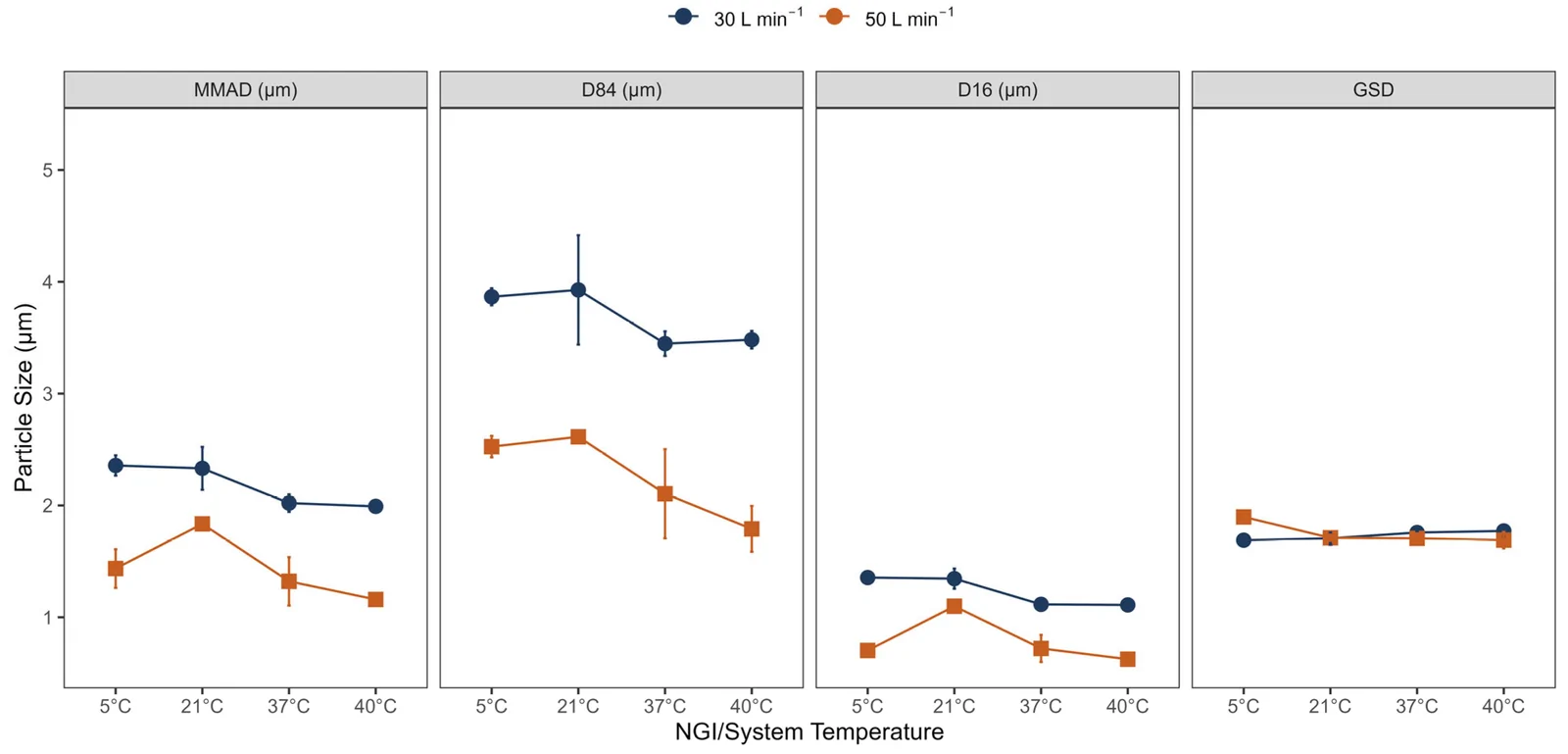
**Effect of NGI/system temperature and NHF on aerodynamic particle-size descriptors for the commercial isotonic salbutamol formulation.** Mean MMAD, D84, D16, and GSD are shown for the commercial isotonic salbutamol formulation at NHF rates of 30 and 50 L·min^−1^ across NGI/system temperatures of 5, 21, 37, and 40 °C. Error bars represent standard deviation (SD) (*n* = 3). Increasing NGI/system temperature was generally associated with reductions in MMAD, D84, and D16 under both NHF conditions. The magnitude of these changes was greater at 50 L·min^−1^ than at 30 L·min^−1^. In contrast, GSD remained within a relatively narrow range across the experimental matrix, although condition-specific changes in distribution breadth were evident.

**Figure 3:**
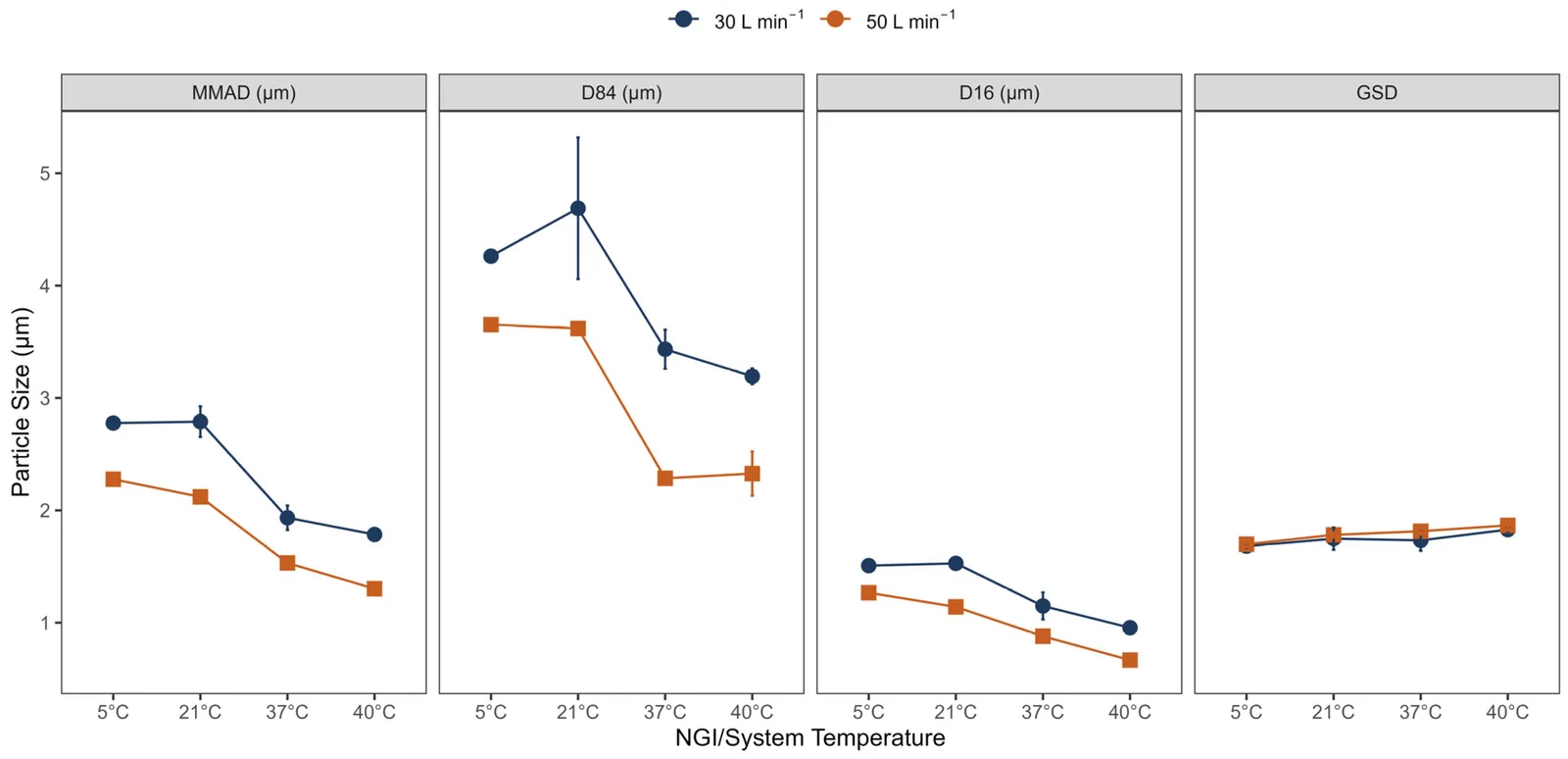
**Effect of NGI/system temperature and NHF on aerodynamic particle-size descriptors for the 3% hypertonic salbutamol formulation.** Mean MMAD, D84, D16, and GSD are shown for the 3% HSS formulation at NHF rates of 30 and 50 L·min^−1^ across NGI/system temperatures of 5, 21, 37, and 40 °C. Error bars represent standard deviation (SD) (*n* = 3). Increasing NGI/system temperature was associated with progressive reductions in MMAD, D84, and D16 under both NHF conditions, indicating a shift in the aerosol size distribution toward smaller aerodynamic diameters. The magnitude of these changes was generally greater than that observed for the ISS formulation, particularly for MMAD. In contrast, GSD varied only modestly across the experimental matrix, suggesting that NGI/system temperature primarily translated the particle-size distribution toward smaller sizes rather than substantially altering distribution breadth.

**Figure 4:**
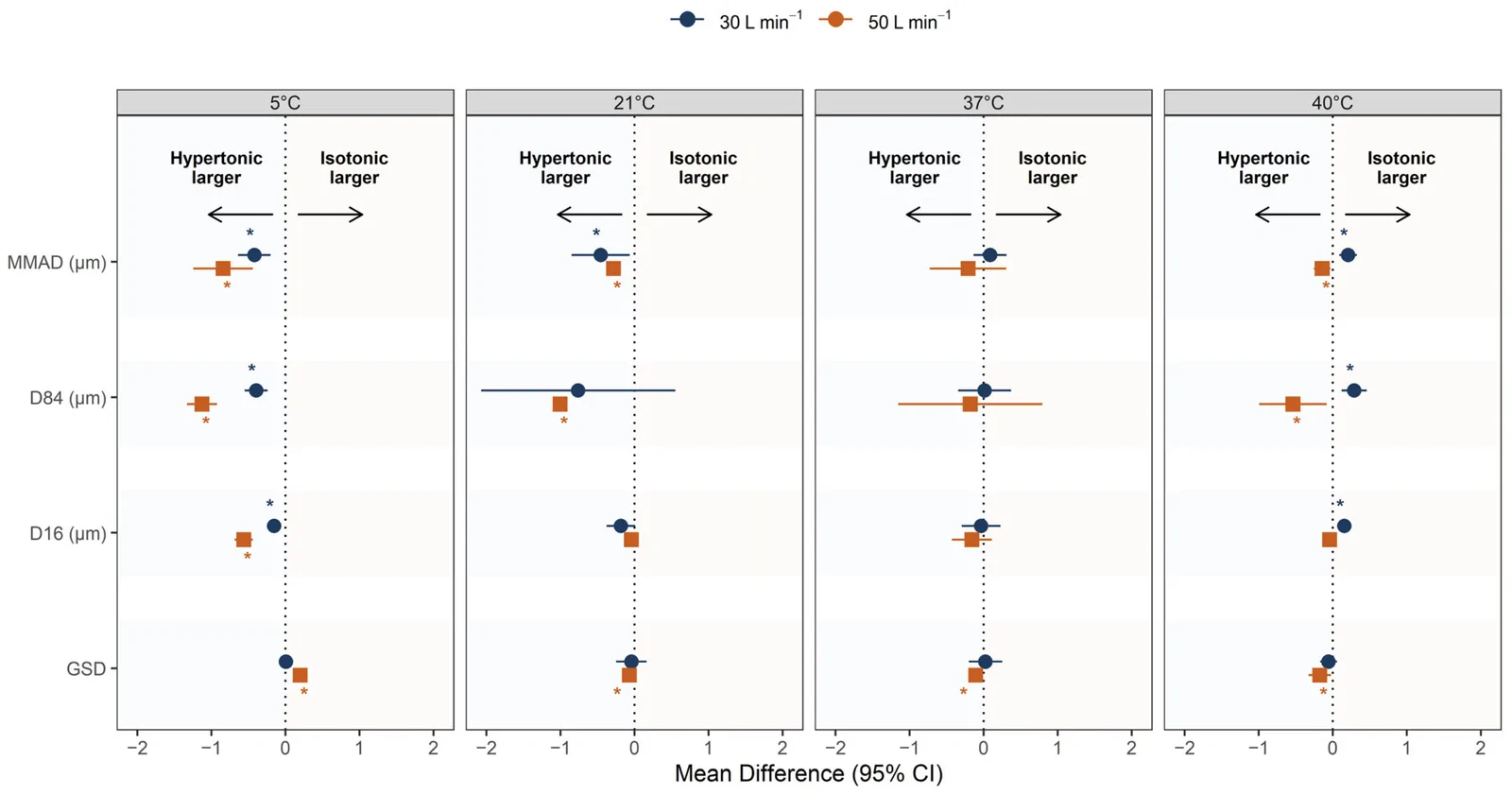
**Matched isotonic-versus-hypertonic formulation contrasts for aerodynamic particle-size descriptors at different NGI/system temperatures and NHF rates.** Points represent the mean difference between formulations (Isotonic–Hypertonic), with horizontal error bars indicating the 95% confidence interval of the difference. Results are shown separately for NGI/system temperatures of 5, 21, 37, and 40 °C and for NHF rates of 30 and 50 L·min^−1^. The vertical dashed line denotes zero difference between formulations. Positive values indicate larger values for the ISS formulation, whereas negative values indicate larger values for the 3% HSS formulation. Comparisons are shown for MMAD, D84, D16, and GSD. At 5 and 21 °C, the 3% HSS formulation generally exhibited larger MMAD, D84, and D16 values than the ISS formulation, particularly at 50 L·min^−1^, indicating a larger aerodynamic size distribution under cooler NGI/system conditions. As NGI/system temperature increased, these formulation-related differences progressively narrowed and, for some particle-size descriptors, approached convergence or reversed direction. In contrast, GSD exhibited comparatively smaller and less consistent formulation-dependent differences across the experimental matrix. Asterisks indicate matched ISS-versus-3% HSS contrasts that remained statistically significant following Benjamini–Hochberg false discovery rate correction (q < 0.05), and colors correspond to the associated NHF rate.

**Figure 5:**
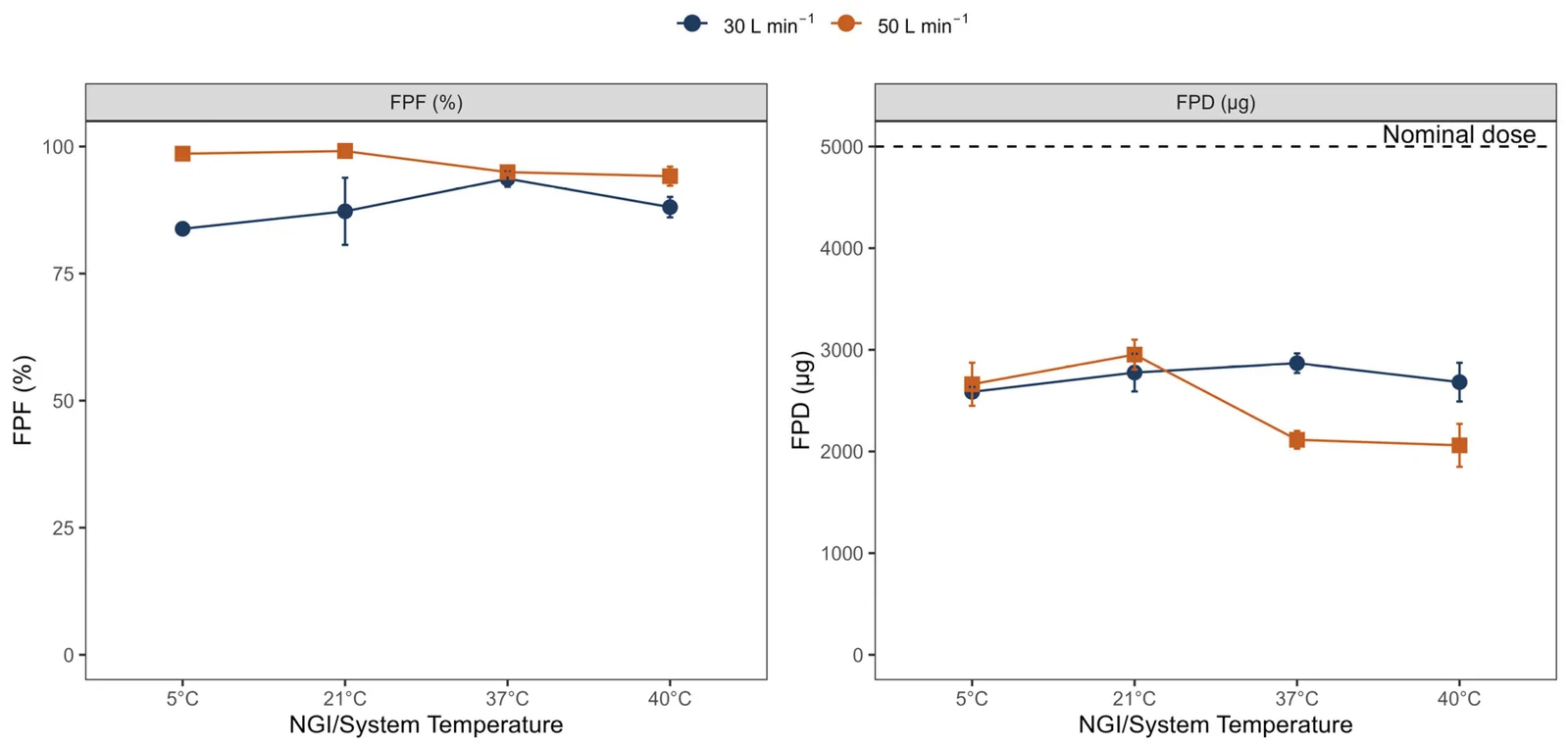
**Effect of NGI/system temperature and NHF on Fine Particle Fraction (FPF) and Fine Particle Dose (FPD) for the commercial isotonic salbutamol formulation.** Mean FPF (%) (**left panel**) and FPD (µg) (**right panel**) are shown following NHF-conditioned transport at NHF rates of 30 and 50 L·min^−1^ across NGI/system temperatures of 5, 21, 37, and 40 °C. FPF represents the proportion of recovered aerosol contained within the respirable size range (<5 µm aerodynamic diameter), whereas FPD represents the corresponding respirable aerosol mass recovered downstream of NHF-conditioned transport. Error bars represent standard deviation (SD) (*n* = 3). The dashed horizontal line in the FPD panel indicates the nominal loaded dose (5000 µg). At higher NGI/system temperatures (37–40 °C), FPD decreased substantially at 50 L·min^−1^ despite comparable or higher FPF values, illustrating that improvements in respirable fraction were not necessarily accompanied by increased delivered respirable mass.

**Figure 6:**
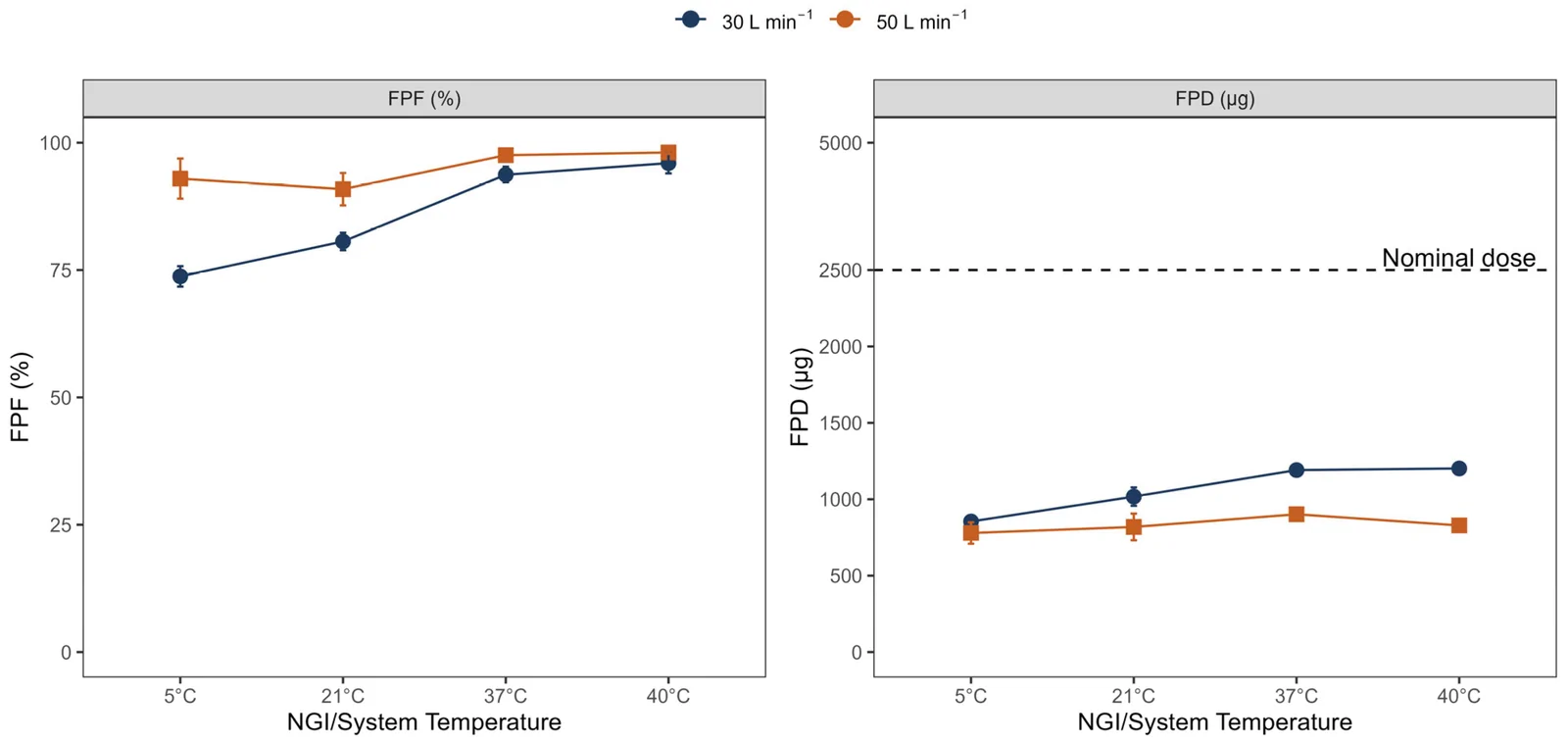
**Effect of NGI/system temperature and NHF on Fine Particle Fraction (FPF) and Fine Particle Dose (FPD) for the 3% hypertonic salbutamol formulation.** Mean FPF (%) (**left panel**) and FPD (µg) (**right panel**) are shown following NHF-conditioned transport at NHF rates of 30 and 50 L·min^−1^ across NGI/system temperatures of 5, 21, 37, and 40 °C. FPF represents the proportion of recovered aerosol contained within the respirable size range (<5 µm aerodynamic diameter), whereas FPD represents the corresponding respirable aerosol mass recovered downstream of NHF-conditioned transport. Error bars represent standard deviation (SD) (*n* = 3). The dashed horizontal line in the FPD panel indicates the nominal loaded dose (2500 µg). Despite consistently higher FPF at 50 L·min^−1^, FPD remained lower than at 30 L·min^−1^ across the experimental matrix, indicating that increases in respirable fraction did not necessarily translate into equivalent increases in delivered respirable aerosol mass.

**Figure 7:**
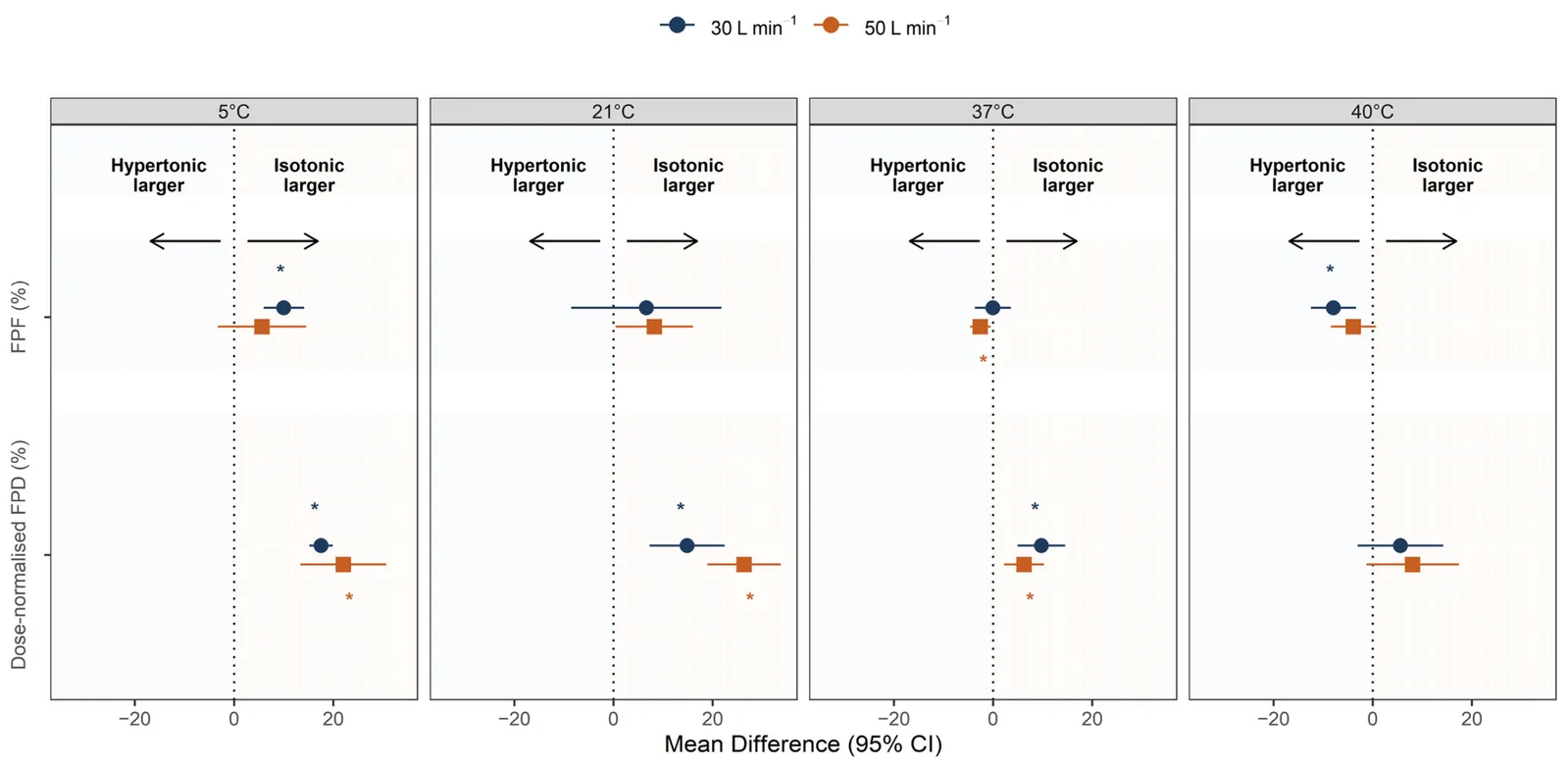
**Matched isotonic-versus-hypertonic formulation contrasts for Fine Particle Fraction (FPF) and dose-normalized Fine Particle Dose (FPD) at different NGI/system temperatures and NHF rates.** Points represent the mean difference between formulations (Isotonic–Hypertonic), with horizontal error bars indicating the 95% confidence interval of the difference. Results are shown separately for NGI/system temperatures of 5, 21, 37, and 40 °C and for NHF rates of 30 and 50 L·min^−1^. The vertical dashed line denotes zero difference between formulations. Positive values indicate higher responses for the ISS formulation, whereas negative values indicate higher responses for the 3% HSS formulation. FPF comparisons are shown in the upper panels and dose-normalized FPD comparisons in the lower panels. The figure demonstrates that formulation effects were highly dependent on the downstream thermal and flow environment. At lower NGI/system temperatures, the ISS generally exhibited higher FPF and dose-normalized FPD, whereas several FPF contrasts converged or reversed at higher temperatures. In contrast, dose-normalized FPD remained consistently higher for the ISS formulation across most conditions. Asterisks indicate matched ISS-versus-3% HSS contrasts that remained statistically significant following Benjamini–Hochberg false discovery rate correction (q < 0.05), and colors correspond to the associated NHF rate.

**Figure 8:**
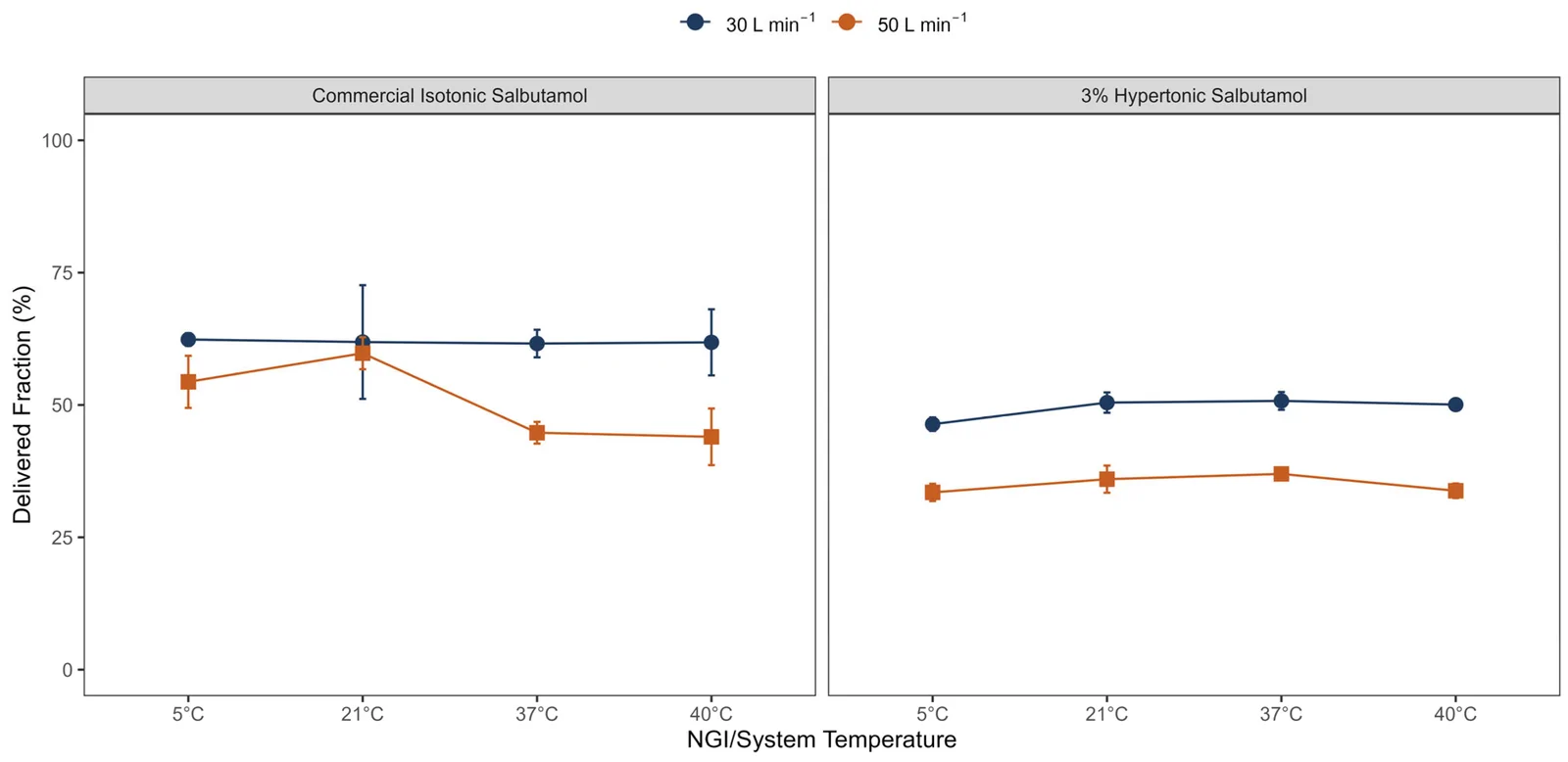
**Delivered Fraction (DF) following NHF-conditioned aerosol transport at different NGI/system temperatures and NHF rates.** Mean DF is shown for the ISS formulation (**left panel**) and the 3% HSS formulation (**right panel**) at NHF rates of 30 and 50 L·min^−1^ across NGI/system temperatures of 5, 21, 37, and 40 °C. DF represents the proportion of the loaded dose recovered downstream of the NHF pathway. Error bars represent standard deviation (SD) (*n* = 3). Across both formulations, increasing NHF generally reduced DF, indicating greater aerosol losses within the NHF pathway. Temperature-dependent changes were modest for the ISS formulation at 30 L·min^−1^ but became more apparent at 50 L·min^−1^, whereas the 3% HSS formulation exhibited lower DF across the experimental matrix. The figure illustrates the combined influence of NHF, formulation, and NGI/system temperature on downstream aerosol recovery following NHF-conditioned transport.

**Figure 9:**
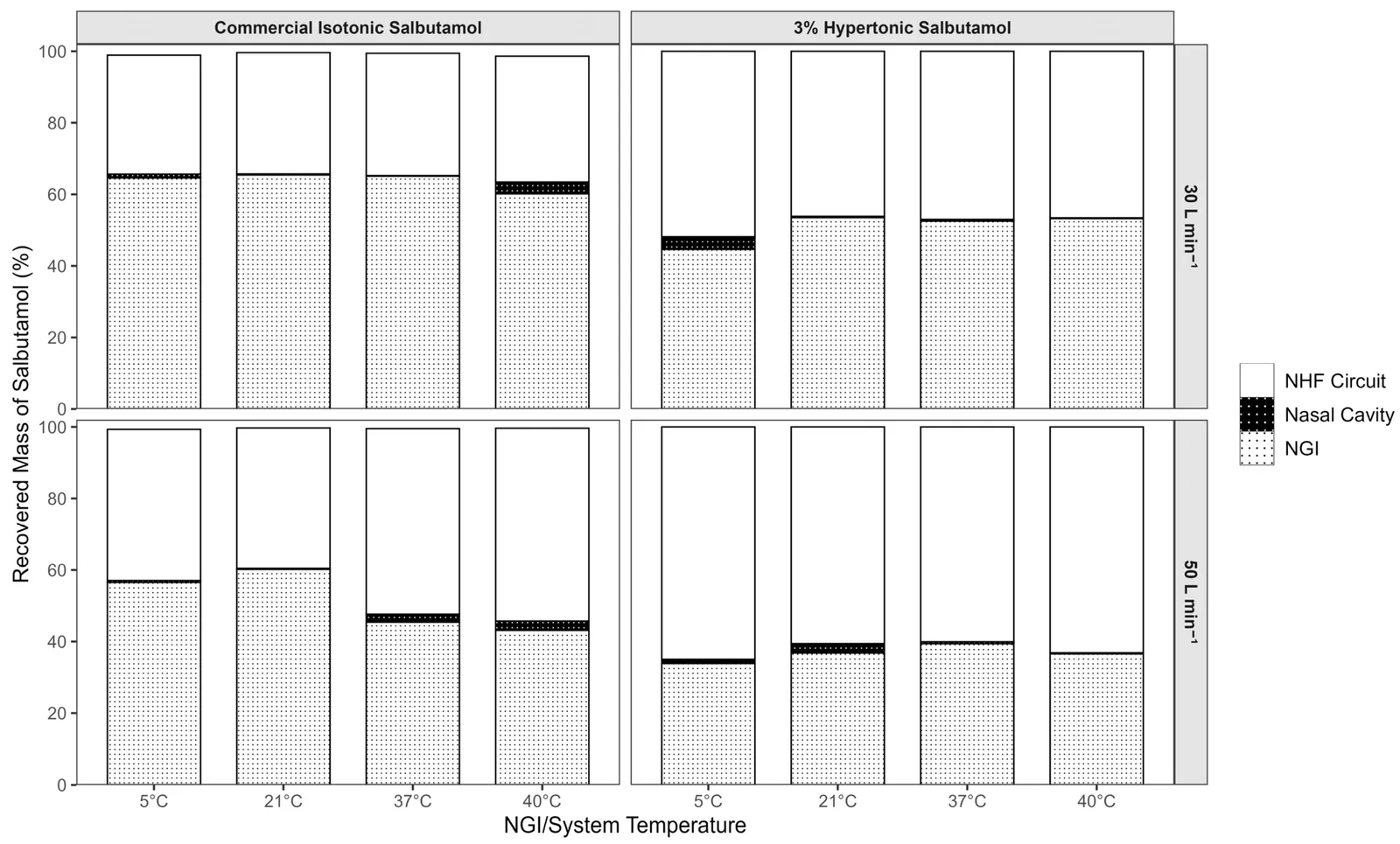
**Regional redistribution of recovered salbutamol across the NHF-to-NGI pathway at different downstream NGI/system temperatures and NHF rates.** Stacked bars show the mean regional partitioning of recovered salbutamol mass for the commercial isotonic salbutamol formulation (left column) and the 3% HSS formulation (right column) at 30 and 50 L min^−1^ (top and bottom rows, respectively) across downstream NGI/system temperatures of 5, 21, 37, and 40 °C. Recovered salbutamol is grouped into three plotted regions: NHF circuit deposition, nasal cavity deposition, and NGI deposition. NHF circuit deposition represents drug retained within the NHF delivery pathway upstream of the nasal replica. NGI deposition represents the cumulative fraction recovered on NGI stages S1–S7 and the combined MOC/filter collection stage, corresponding to aerosol available for downstream penetration beyond the nasal airway model under continuous-entrainment conditions. Corresponding regional deposition results expressed as a percentage of nominal loaded dose, together with individual NGI-stage deposition data, are provided in Supplementary Material S11.

**Figure 10:**
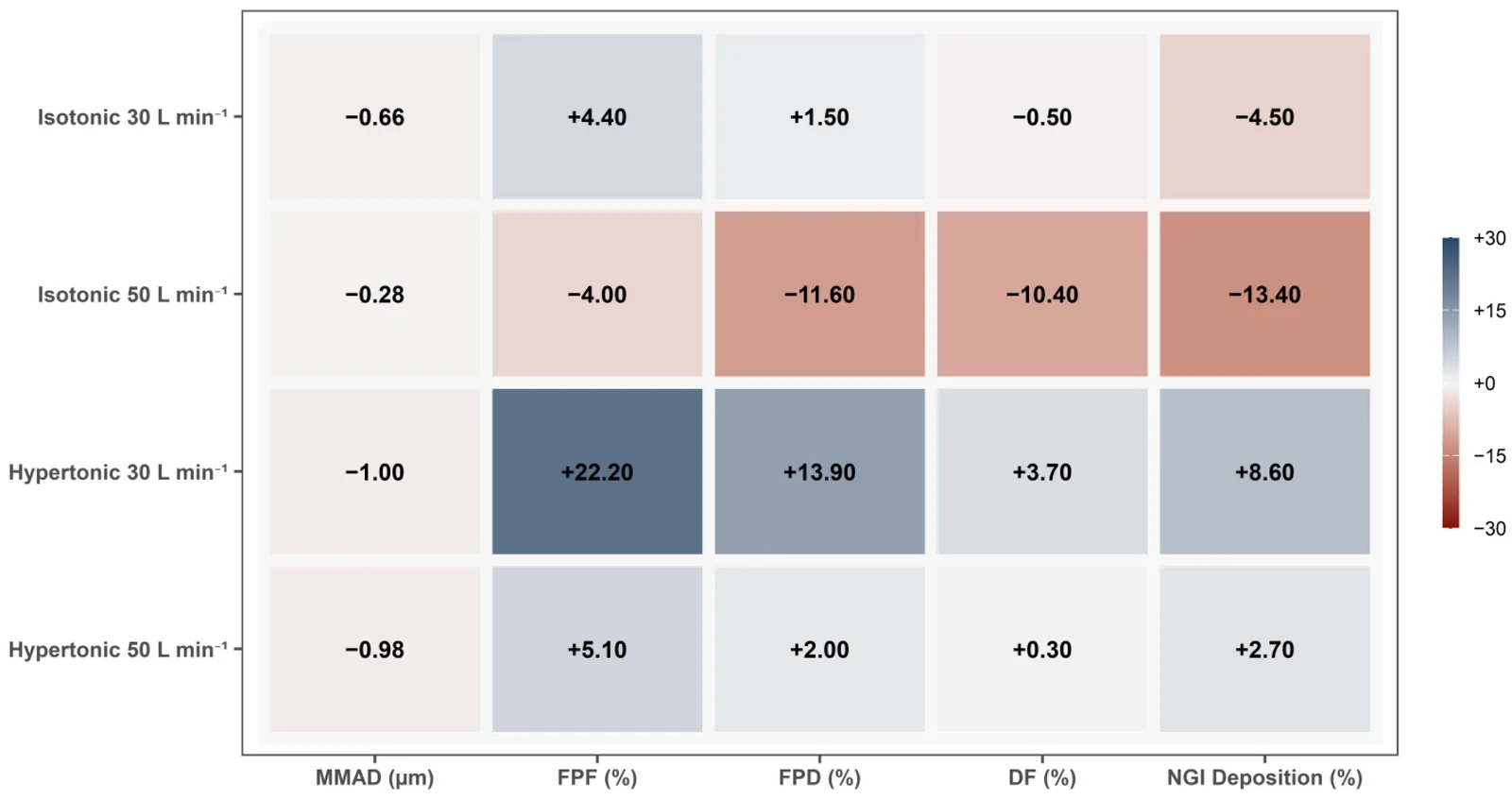
**Similar MMAD responses produce different fine particle, delivery, and deposition outcomes.** Response magnitudes were calculated as the change between conventional cooled (5 °C) and closer to body temperature (37 °C) NGI/system conditions for each formulation and NHF-rate combination. The figure compares the corresponding changes in aerodynamic particle size (MMAD), fine-particle fraction (FPF), dose-normalized fine-particle dose (FPD), NHF delivery efficiency as delivered fraction (DF), and NGI deposition. Despite comparable reductions in MMAD under several conditions, the magnitude and direction of the associated changes in FPF, FPD, DF, and NGI deposition varied substantially.

**Table 1:** Effects of NGI/system temperature and NHF rate on particle-size metrics for the ISS formulation.

Particle-Size Metrics	Temperature *p*	Temperature η^2^	Flow *p*	Flow η^2^	Temp × Flow *p*	Temp × Flow η^2^
MMAD	<0.0001 *	0.7798	<0.0001 *	0.9248	0.0570	0.3666
D84	0.0006 *	0.6484	<0.0001 *	0.9277	0.4983	0.1342
D16	<0.0001 *	0.8736	<0.0001 *	0.9486	0.0004 *	0.6685
GSD	0.0219 *	0.4430	0.2813	0.0721	0.0001 *	0.7187

Type III ANOVA results for within-formulation analysis (Response ~ Temperature × Flow). Reported values are *p*-values and partial η^2^ effect sizes. Statistically significant effects (*p* < 0.05) are indicated by *. Complete ANOVA outputs, including degrees of freedom and F-statistics, are provided in Supplementary S7.

**Table 2:** Effects of NGI/system temperature and NHF rate on particle-size metrics for the 3% HSS formulation.

Particle-Size Metrics	Temperature *p*	Temperature η^2^	Flow *p*	Flow η^2^	Temp × Flow *p*	Temp × Flow η^2^
MMAD	<0.0001 *	0.9830	<0.0001 *	0.9532	0.0292 *	0.4213
D84	<0.0001 *	0.9079	<0.0001 *	0.8420	0.2646	0.2142
D16	<0.0001 *	0.9668	<0.0001 *	0.9199	0.1384	0.2841
GSD	0.0012 *	0.6191	0.0695	0.1913	0.7626	0.0680

Type III ANOVA results for within-formulation analysis (Response ~ Temperature × Flow). Reported values are *p*-values and partial η^2^ effect sizes. Statistically significant effects (*p* < 0.05) are indicated by *. Complete ANOVA outputs, including degrees of freedom and F-statistics, are provided in Supplementary S7.

## Data Availability

The data presented in this study are available within the article and its Supplementary Materials. Additional data are available from the corresponding author upon request.

## Supplementary Materials

The following supporting information can be downloaded at: https://www.mdpi.com/article/10.3390/pharmaceutics18101234/s1, S1: Calculation of NGI Stage Cut-off Diameters at 50 L/min; S2: APSD Calculations and Aerosol Performance Endpoint Definitions; S3: Full DOE Randomization, Run Orders, Factors, Responses, and Results; S4: UV Spectrophotometric Quantification of Salbutamol; S5: Recovery of Salbutamol from Individual Experimental Runs; S6: Statistical Analysis; S7: Full Factorial ANOVA Results; S8: Welch Pairwise Contrasts; S9: Model Diagnostics; S10: False Discovery Rate (FDR) Sensitivity Analysis; S11: Regional and Individual NGI-Stage Deposition Expressed as Percentage of Nominal Loaded Dose; and S12: Pre-Device Drug Loss (% Nominal Loaded Dose).

## Abbreviations

The following abbreviations are used in this manuscript:

ISS	Commercial Isotonic Salbutamol Sulfate Formulation
3% HSS	3% Hypertonic Salbutamol Sulfate Formulation
ANOVA	Analysis of Variance
APSD	Aerodynamic Particle-Size Distribution
CI	Confidence Interval
DF	Delivered Fraction
DOE	Design of Experiments
D16	16th Percentile Aerodynamic Diameter
D84	84th Percentile Aerodynamic Diameter
FPD	Fine Particle Dose
FPF	Fine Particle Fraction
GSD	Geometric Standard Deviation
HFNC	High-Flow Nasal Cannula
HFOT	High-Flow Oxygen Therapy
MMAD	Mass Median Aerodynamic Diameter
MOC	Micro-Orifice Collector
NaCl	Sodium Chloride
NGI	Next Generation Impactor
NHF	Nasal High-Flow
NHF-to-NGI	Nasal High-Flow to Next Generation Impactor
RH	Relative Humidity
SD	Standard Deviation
T × F	Temperature × Flow Interaction
UV	Ultraviolet
