# Supplementary material for: Nebulized Aerosol Characteristics During Nasal High-Flow: Effect of Flow, Formulation and Impactor Temperature
Source: Pharmaceutics. 2026 Sep 29;18(10):1234. doi: 10.3390/pharmaceutics18101234 (PMC13708300; doi:10.3390/pharmaceutics18101234)
Supplement: Supplementary file 1 [file pharmaceutics-18-01234-s001.zip › pharmaceutics-4546029-supplementary.pdf]

Supplementary Materials

S1. Calculation of NGI Stage Cut-off Diameters at 50 L/min

For operation of the Next Generation Impactor (NGI) at 50 L min−1, stage cut-off diameters were calculated using the archival NGI calibration model rather than by linear interpolation. The NGI was originally calibrated at 30, 60, and 100 L min−1, and these data were used to develop equations that predict the particle cut size for all impactor components over the range 30–100 L min−1. This approach is consistent with inertial impaction physics, in which stage cut-off diameter is governed by Stokes number and nozzle-exit velocity and therefore varies nonlinearly with flow rate.

For each NGI stage i, the cut-off diameter at flow Q was calculated using the empirical archival-calibration relationship:

$$D_{50,i}(Q) = A_i\ (Q/60)^{-B_i}$$

where $D_{50,i}(Q)$ is the aerodynamic cut-off diameter for stage i at flow Q (μm), Q is flow rate (L min−1), and Ai and Bi are stage-specific empirical constants for the 30–100 L min−1 archival calibration range.

The stage-specific constants used were: Stage 1, A = 8.06 and B = 0.54; Stage 2, A = 4.46 and B = 0.52; Stage 3, A = 2.82 and B = 0.50; Stage 4, A = 1.66 and B = 0.47; Stage 5, A = 0.94 and B = 0.53; Stage 6, A = 0.55 and B = 0.60; and Stage 7, A = 0.34 and B = 0.67.

Table S1. NGI cut-off diameters at 30, 50, and 60 L min−1.

NGI location	30 L min^{-1} cut-off (μm)	50 L min^{-1} calculated cut-off (μm)	60 L min^{-1} cut-off (μm)
Stage 1	11.72	8.90	8.06
Stage 2	6.40	4.90	4.46
Stage 3	3.99	3.09	2.82
Stage 4	2.30	1.81	1.66
Stage 5	1.36	1.04	0.94
Stage 6	0.83	0.61	0.55
Stage 7	0.54	0.38	0.34

CONFIDENTIAL

S2. APSD Calculations and Aerosol Performance Endpoint Definitions

MMAD, D16, and D84 were calculated from cumulative aerodynamic particle-size distributions generated from NGI stage deposition data. The target cumulative mass fractions corresponding to the 16th, 50th, and 84th percentiles were identified, and the corresponding aerodynamic diameters were determined by linear interpolation between the two neighbouring stage data points bounding the target cumulative mass fraction.

GSD was subsequently calculated as:

$$GSD = \sqrt{\frac{D84}{D16}}$$

APSD calculations incorporated deposition recovered from NGI stages S1-S7 together with the terminal MOC/filter collection stage. The filter paper located within the MOC collection stage was recovered and analysed together with the MOC as a single terminal collection location.

Table S2. Definitions and Calculation Methods for Aerosol Performance Endpoints.

**Endpoint**	**Definition / Calculation**	**Numerator**	**Denominator**	**Unit**
FPF (%)	Fine particle fraction of delivered aerosol	Fine particle mass (<5 µm aerodynamic diameter) recovered within the NGI	Total NGI-recovered mass (S1-S7 + MOC/filter) +Mass recovered from nasal cavity	%
FPD (µg)	Fine particle dose recovered downstream of NHF transport	Fine particle mass (<5 µm aerodynamic diameter) recovered within the NGI	Not applicable	µg
Dose-normalised FPD (%)	Fine particle dose normalised to nominal loaded dose	FPD	Nominal loaded dose	%
Delivered Fraction (DF, %)	Downstream delivery efficiency	Total mass recovered in Nasal Cavity + NGI stages + MOC/filter	Loaded dose for ISS (Nominal dose−Pre-device drug loss) and Nominal loaded dose for 3% HSS (as there is	%

			no pre-device drug loss by design)	
NHF Circuit Deposition (%)	Regional deposition endpoint reported on recovered-mass basis	NHF circuit recovered mass (Device, Chamber, Breathing tube, and Cannula)	Total recovered mass	%
Nasal-Cavity Deposition (%)	Regional deposition endpoint reported on recovered-mass basis	Nasal replica recovered mass	Total recovered mass	%
NGI Deposition (%)	Regional deposition endpoint reported on recovered-mass basis	NGI stages S1-S7 + MOC/filter recovered mass	Total recovered mass	%
Pre-device Drug Loss (%)	Residual drug remaining in the ampoule before entry to the delivery system (ISS only)	Ampoule recovered mass	Nominal loaded dose	%

Terms such as delivery efficiency, downstream aerosol delivery are used descriptively throughout the manuscript to refer to aerosol mass recovered downstream of the NHF circuit. In the present study, formal statistical analyses were performed using the explicitly defined endpoints listed in Table S2. NGI deposition refers specifically to mass recovered on NGI stages S1–S7 together with the combined MOC/filter collection stage. Regional deposition results were reported separately for the NHF circuit, nasal cavity, and NGI compartments.

CONFIDENTIAL

S3. Full DOE Design, Randomization, Experimental Conduct and Results

All experiments were performed using the same Airvo™ 3 device and the same nasal airway replica throughout the study. A new combination of NHF accessories (humidification chamber, heated breathing tube, and cannula) was used for each experimental run. Five Aerogen Solo vibrating mesh nebulisers were randomly assigned across experimental conditions. Nebulizer identity was not evaluated as an experimental factor. The experimental programme was conducted over a period of approximately three months. The experimental runs were completed according to the randomized run order reported in Table S3a.

Following each experiment, all reusable components (nasal cavity, NHF device) were thoroughly cleaned extensively with distilled water as required. The nebuliser was rinsed extensively and air dried before reuse. Visual inspection was performed after cleaning to ensure removal of residual deposits prior to subsequent use.

Table S3a. Full DOE Design and results table.

Replicate	Run Order	Std Run	Temp (°C)	Tonicity	Flow (L min^{-1})	MMAD (μm)	GSD	FPD (μg)	FPF (%)	Delivered Fraction (%)	d16	d84
1	1	11	37	ISS	50	1.268	1.746	2083.381	94.777	44.143	0.689	2.100
1	2	2	21	ISS	30	2.552	1.761	2942.770	79.798	74.097	1.448	4.490
1	3	4	40	ISS	30	2.027	1.719	2509.976	88.485	57.218	1.157	3.418
1	4	10	21	ISS	50	1.792	1.736	2854.601	99.450	57.671	1.067	2.604
1	5	1	5	ISS	30	2.279	1.684	2578.615	82.570	63.607	1.339	3.794
1	6	8	40	3% HSS	30	1.851	1.812	1198.204	94.672	50.625	0.991	3.255
1	7	9	5	ISS	50	1.635	1.852	2902.770	97.369	60.032	0.769	2.636
1	8	15	37	3% HSS	50	1.507	1.827	904.896	97.464	37.138	0.856	2.249
1	9	14	21	3% HSS	50	2.122	1.758	757.500	89.176	33.978	1.185	3.664
1	10	3	37	ISS	30	2.087	1.750	2833.119	92.045	61.972	1.154	3.537
1	11	5	5	3% HSS	30	2.779	1.693	852.326	74.775	45.594	1.507	4.319
1	12	16	40	3% HSS	50	1.284	1.834	850.493	98.271	34.618	0.658	2.215
1	13	7	37	3% HSS	30	2.057	1.670	1191.736	91.979	52.398	1.276	3.559
1	14	12	40	ISS	50	1.216	1.772	2156.452	94.077	46.097	0.642	2.016
1	15	13	5	3% HSS	50	2.316	1.669	699.410	88.447	31.631	1.326	3.692
1	16	6	21	3% HSS	30	2.652	1.644	1078.090	82.162	52.486	1.492	4.032
2	1	7	37	3% HSS	30	1.895	1.839	1192.535	95.073	49.038	1.037	3.507
2	2	15	37	3% HSS	50	1.575	1.782	889.201	98.595	36.075	0.925	2.338
2	3	11	37	ISS	50	1.560	1.711	2047.952	95.643	43.083	0.855	2.505
2	4	2	21	ISS	30	2.208	1.695	2576.526	89.580	57.655	1.282	3.684
2	5	10	21	ISS	50	1.859	1.684	3121.549	98.924	63.248	1.127	2.607
2	6	1	5	ISS	30	2.337	1.702	2637.676	85.032	62.279	1.333	3.859
2	7	9	5	ISS	50	1.328	1.927	2574.366	99.641	52.010	0.664	2.464
2	8	12	40	ISS	50	1.108	1.629	2205.833	92.324	47.954	0.610	1.617
2	9	8	40	3% HSS	30	1.744	1.826	1230.423	98.293	50.072	0.935	3.117
2	10	13	5	3% HSS	50	2.274	1.717	807.153	94.685	34.099	1.240	3.655

2	11	3	37	ISS	30	2.040	1.757	2977.500	93.695	64.009	1.126	3.479
2	12	16	40	3% HSS	50	1.350	1.919	787.148	97.787	32.199	0.694	2.554
2	13	4	40	ISS	30	1.974	1.822	2886.476	85.888	68.927	1.076	3.569
2	14	5	5	3% HSS	30	2.786	1.667	854.410	71.447	47.835	1.516	4.211
2	15	14	21	3% HSS	50	2.138	1.803	918.785	94.541	38.874	1.111	3.612
2	16	6	21	3% HSS	30	2.925	1.840	958.229	78.761	48.665	1.561	5.288
3	1	2	21	ISS	30	2.235	1.662	2809.024	92.365	53.913	1.306	3.608
3	2	11	37	ISS	50	1.137	1.660	2213.548	94.443	47.042	0.620	1.708
3	3	3	37	ISS	30	1.935	1.766	2795.000	95.253	58.831	1.065	3.325
3	4	6	21	3% HSS	30	2.790	1.761	1015.486	80.913	50.201	1.531	4.746
3	5	16	40	3% HSS	50	1.275	1.846	848.542	98.208	34.561	0.649	2.213
3	6	14	21	3% HSS	50	2.100	1.783	780.174	88.912	35.099	1.126	3.580
3	7	9	5	ISS	50	1.345	1.913	2506.648	98.760	51.076	0.677	2.478
3	8	1	5	ISS	30	2.456	1.683	2541.976	83.816	61.216	1.393	3.945
3	9	8	40	3% HSS	30	1.760	1.847	1175.903	94.993	49.515	0.940	3.208
3	10	13	5	3% HSS	50	2.240	1.711	830.833	95.731	34.715	1.234	3.612
3	11	12	40	ISS	50	1.153	1.669	1818.214	96.084	37.899	0.623	1.736
3	12	7	37	3% HSS	30	1.850	1.689	1188.507	94.093	50.878	1.134	3.234
3	13	5	5	3% HSS	30	2.765	1.684	856.944	75.043	45.678	1.501	4.256
3	14	15	37	3% HSS	50	1.513	1.833	911.042	96.591	37.728	0.858	2.268
3	15	4	40	ISS	30	1.974	1.775	2649.738	89.839	59.342	1.098	3.459
3	16	10	21	ISS	50	1.854	1.713	2883.516	98.938	58.404	1.102	2.630

The Table S3a presents replicate-level data for the principal endpoints reported in the manuscript. Dose-normalised FPD is not presented as a separate column because it was derived during statistical analysis in R by normalising absolute FPD to the formulation-specific nominal loaded dose. Additional exploratory endpoints, including FPF Loaded, FPF Recovered, dose-normalised Delivered Dose, and absolute Delivered Dose, were retained in the complete analysis dataset and included in the FDR sensitivity analysis but are not tabulated because they were not used to support the primary study findings.

Rejection and Repetition of runs

A total of seven experimental runs were rejected and repeated. Six runs were rejected because impaired water delivery from the NHF system resulted in complete evaporation and crystalline residue in the NGI collection cups. The criterion concerning complete evaporation and crystalline residue was established during preliminary method development before commencement of the formal experimental runs. One additional run, involving the 3% HSS formulation at 21 °C and 50 L·min^{-1}, was rejected because it exhibited a visibly distorted NGI deposition pattern. This abnormality was first identified during the experimental programme and was not covered by the original exclusion criterion. Following this event, a visibly distorted NGI deposition pattern was added as an exclusion criterion for subsequent runs.

The affected NGI was completely disassembled, inspected, and reassembled, and the abnormal deposition pattern did not recur when the condition was repeated. All exclusion decisions were based on observable experimental abnormalities identified before the affected runs were accepted into the final analysis and were not based on the magnitude, direction, or statistical significance of the measured aerosol-performance outcomes. All seven runs were repeated successfully, only the valid replacement runs were included in the final 48-run analysis, and every combination retained the planned three valid replicates. The condition-specific distribution and reasons for rejection are reported in Table S3b.

Table S3b. Distribution and disposition of rejected and repeated experimental runs.

Replicate	Run Order	Std Run*	Temp (°C)	Tonicity	Flow (L min^{-1})	Reason for Repetition
1	1	11	37	ISS	50	Crystalline residue collected in NGI cups
1	2	2	21	ISS	30	
1	6	8	40	3% HSS	30	
1	10	3	37	ISS	30	
1	16	6	21	3% HSS	30	
3	4	6	21	3% HSS	30	
2	15	14	21	3% HSS	50	Visually distorted NGI deposition pattern

*All rejected runs were repeated; valid replacement run included in the final analysis.

CONFIDENTIAL

S4. UV Spectrophotometric Quantification of Salbutamol

Salbutamol sulfate was quantified using UV spectrophotometry at 276 nm (Specord 250 Plus, Analytik Jena, Germany). Calibration standards were prepared by serial dilution of the corresponding study formulation in distilled water. The calibration ranges were 0.78–200 μg·mL^{-1} for ISS and 0.39–100 μg·mL^{-1} for 3% HSS. Three independent calibration series were generated on separate analytical days for each formulation. The slope, intercept, and coefficient of determination (R^2) for each calibration series are reported in Table S4a. A fresh calibration curve was also prepared during each analytical session and used to quantify the samples analysed during that session. Representative ISS and 3% HSS calibration curves are shown in Figures S1a and S1b, respectively.

Table S4a. Regression parameters for independent salbutamol calibration series.

**Formulation**	**Analytical day**	**Calibration range (μg·mL^{-1})**	**Slope**	**Intercept**	**R^2**
ISS	1	0.78–200	0.007106	−0.003070	0.999973
ISS	2	0.78–200	0.007005	0.001408	0.999984
ISS	3	0.78–200	0.007127	0.006231	0.999860
3% HSS	1	0.39–100	0.007172	0.002957	0.999969
3% HSS	2	0.39–100	0.007094	0.001934	0.999952
3% HSS	3	0.39–100	0.007135	0.002441	0.999974

Inter-day consistency of the calibration response was assessed from the variability in calibration slopes across the three independent analytical days. The mean slope, slope RSD, and mean R^2 values are provided in Table S4b. The results indicated consistent calibration slopes and linear responses across the analytical days evaluated. A separate intraday precision study was not performed.

Table S4b. Inter-day consistency of calibration responses.

**Formulation**	**Independent analytical days**	**Mean slope**	**SD of slope**	**Slope RSD (%)**	**Mean R^2**
ISS	3	0.0070793	0.0000652	0.92	0.999939
3% HSS	3	0.0071337	0.0000390	0.55	0.999965

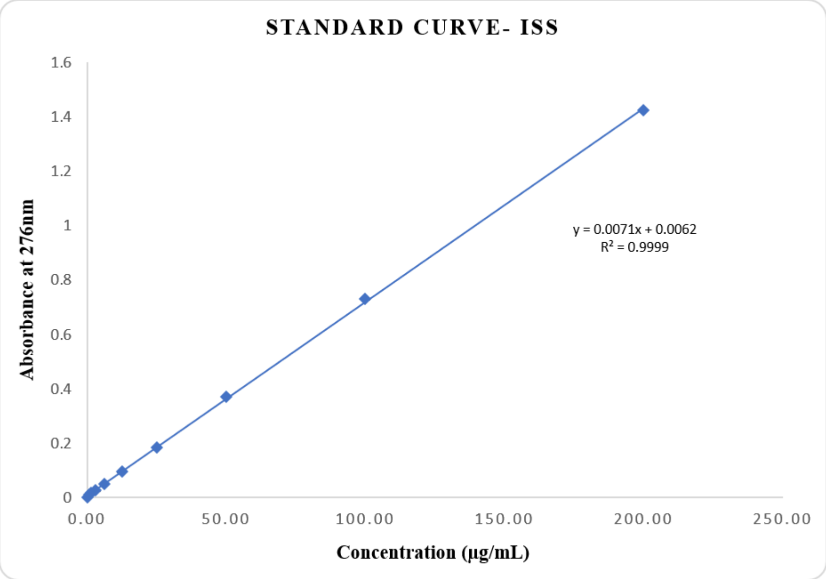

Figure S1a. Representative calibration curve for the commercial isotonic salbutamol formulation. Calibration standards were prepared by serial dilution of the formulation over the concentration range 0.78–200 µg·mL⁻¹ and analysed at 276 nm. The regression equation and coefficient of determination (R^2) are shown on the figure.

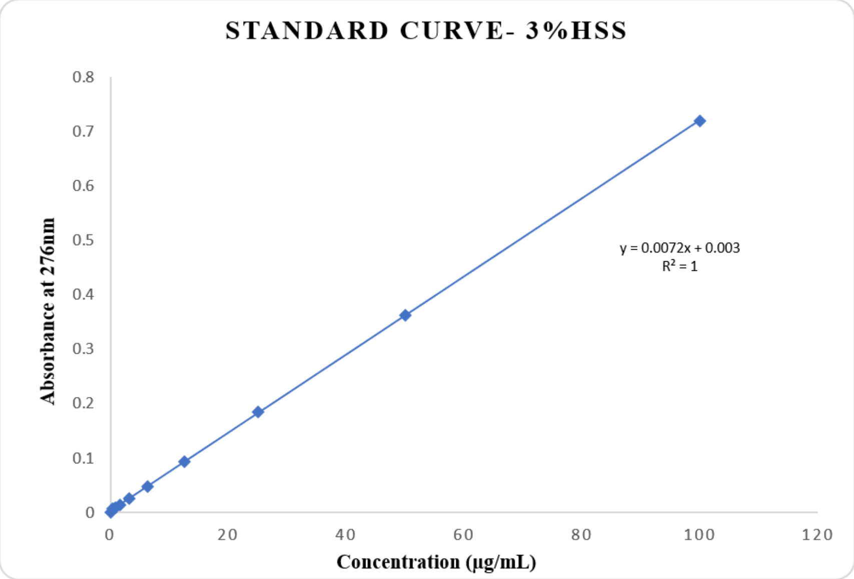

Figure S1b. Representative calibration curve for the hypertonic salbutamol formulation (3% NaCl). Calibration standards were prepared by serial dilution of the formulation over the concentration range 0.39–100 µg·mL⁻¹ and analysed at 276 nm. The regression equation and coefficient of determination (R^2) are shown on the figure.

Distilled water was used to establish the analytical baseline. Sterile isotonic saline (0.9% NaCl) and the 3% hypertonic saline matrix without salbutamol were analysed as blanks and exhibited zero absorbance at 276 nm. These findings indicate that the saline matrices used in the present study did not contribute detectable absorbance at the analytical wavelength.

All samples recovered from the experimental setup were diluted in distilled water and quantified using the calibration curve generated during the corresponding analytical session. Component-

specific salbutamol recovery experiments were not performed. Consequently, independent analytical accuracy and recovery efficiency were not established for the nebulizer, humidification chamber, heated breathing tube, cannula, nasal replica, NGI collection cups, or combined MOC/filter collection stage. The component-level and regional-deposition results were therefore not adjusted for potential differences in recovery efficiency among component materials or surface geometries.

S5. Recovery of Salbutamol from Individual Experimental Runs

Formulation	Flow rate (L min^{-1})	Temperature (°C)	% Nominal Dose Recovered		
			Run 1	Run 2	Run 3
ISS	30	5	97.684	90.393	94.264
		21	99.162	90.731	102.403
		37	95.976	95.621	90.425
		40	94.304	96.461	97.517
	50	5	100.208	92.736	90.774
		21	94.445	101.350	99.950
		37	92.128	95.753	92.913
		40	94.456	95.393	98.327
HSS	30	5	97.291	96.592	98.327
		21	98.456	90.553	92.134
		37	97.719	95.662	91.824
		40	91.105	95.146	95.013
	50	5	90.507	100.704	95.620
		21	92.153	91.484	90.311
		37	92.252	90.357	95.377
		40	90.767	94.331	90.398

S6. Statistical Analysis

Statistical Software and Contrast Coding

All statistical analyses were performed in R version 4.4.1 (R Foundation for Statistical Computing, Vienna, Austria) using pre-specified scripts developed for this study. The principal analysis packages included tidyverse, car, effectsize, readxl, and openxlsx. Figures were generated using ggplot2 and patchwork. Sum-to-zero contrast coding (contr.sum) was used for factorial ANOVA models, with polynomial contrasts (contr.poly) specified for ordered factors.

Factorial and Pairwise Analyses

The primary analysis employed a full factorial model of the form Response ~ Temperature × Formulation × Flow to quantify main effects and interaction terms associated with NGI/system temperature, formulation, and NHF rate.

To examine within-formulation behaviour, additional formulation-specific models (Response ~ Temperature × Flow) were fitted separately for the ISS formulation and the 3% HSS formulation.

Matched isotonic-versus-hypertonic contrasts were performed at each temperature-flow combination using Welch two-sample t-tests. For matched-condition contrasts, uncertainty was quantified using 95% Welch confidence intervals.

Between-formulation comparisons of fine particle dose (FPD) were performed on a dose-normalised basis to account for different nominal loaded doses (5000 μg for the ISS formulation and 2500 μg for the 3% HSS formulation).

Logit Transformation and Model Reporting

Bounded percentage outcomes (FPF, Delivered Fraction, and dose-normalised FPD) were analysed on the logit scale for inference, whereas descriptive statistics are reported on the original measurement scale. To avoid undefined values during transformation, a boundary-adjustment function was implemented whereby values ≤0% and ≥100% were mapped to 0.0001 and 0.9999, respectively, prior to transformation. No analysed endpoint contained exact values of 0% or 100%; therefore, this adjustment did not affect the reported data.

Factorial results are summarised as Type III ANOVA outputs with associated p-values and partial eta-squared (η^2) effect sizes.

To support mechanistic interpretation of deposition behaviour, stage-wise deposition was collapsed into three physiologically interpretable regions: NHF circuit deposition (nebulizer, humidification chamber, heated breathing tube, and cannula), nasal-cavity deposition, and NGI deposition (NGI stages S1-S7 and combined MOC/filter collection stage). Region-level deposition was analysed using the same factorial structure (Region Deposition ~ Temperature × Formulation × Flow).

All summary data are presented as mean ± standard deviation (SD). Statistical significance was interpreted at $p < 0.05$.

Model Diagnostics and FDR Sensitivity Analysis

Model assumptions were assessed using Shapiro-Wilk tests of residual normality and Levene's tests of homogeneity of variance.

To assess the impact of multiplicity on exploratory pairwise comparisons, a Benjamini-Hochberg false discovery rate (FDR) sensitivity analysis was applied cross all 96 Welch pairwise comparisons. Primary statistical inference remained based on the factorial ANOVA framework, whereas the FDR-adjusted Welch comparisons were used to evaluate the robustness of condition-specific contrasts.

S7. Full Factorial ANOVA Results Table

Endpoint	Effect	df1	df2	F-value	p-value	Partial η^2
MMAD	Temperature	3	32	143.14	1.28×10^{-18}	0.931
MMAD	Formulation	1	32	74.51	7.28×10^{-10}	0.700
MMAD	Flow	1	32	438.26	3.06×10^{-20}	0.932
MMAD	Temperature × Formulation	3	32	25.55	1.26×10^{-8}	0.705
MMAD	Temperature × Flow	3	32	1.46	0.2434	0.121
MMAD	Formulation × Flow	1	32	14.07	0.00070	0.305
MMAD	Temperature × Formulation × Flow	3	32	5.07	0.00550	0.322
D16	Temperature	3	32	146.82	8.77×10^{-19}	0.932
D16	Formulation	1	32	56.75	1.41×10^{-8}	0.639
D16	Flow	1	32	478.53	8.19×10^{-21}	0.937
D16	Temperature × Formulation	3	32	25.67	1.19×10^{-8}	0.706
D16	Temperature × Flow	3	32	3.02	0.04390	0.221
D16	Formulation × Flow	1	32	19.10	0.00012	0.374
D16	Temperature × Formulation × Flow	3	32	11.29	3.28×10^{-5}	0.514
D84	Temperature	3	32	53.92	1.30×10^{-12}	0.835
D84	Formulation	1	32	43.22	2.08×10^{-7}	0.575
D84	Flow	1	32	277.18	2.55×10^{-17}	0.897
D84	Temperature × Formulation	3	32	8.81	0.00021	0.452
D84	Temperature × Flow	3	32	0.94	0.4306	0.081
D84	Formulation × Flow	1	32	12.54	0.00124	0.282
D84	Temperature × Formulation × Flow	3	32	1.34	0.2788	0.112
GSD	Temperature	3	32	2.85	0.0530	0.211

CONFIDENTIAL

GSD	Formulation	1	32	3.90	0.0569	0.109
GSD	Flow	1	32	4.90	0.0341	0.133
GSD	Temperature × Formulation	3	32	10.99	4.06×10^{-5}	0.507
GSD	Temperature × Flow	3	32	4.17	0.0134	0.281
GSD	Formulation × Flow	1	32	0.66	0.4230	0.020
GSD	Temperature × Formulation × Flow	3	32	7.08	0.00089	0.399
FPD	Temperature	3	32	5.92	0.0025	0.357
FPD	Formulation	1	32	328.65	2.16×10^{-18}	0.911
FPD	Flow	1	32	0.62	0.4355	0.019
FPD	Temperature × Formulation	3	32	1.73	0.1803	0.140
FPD	Temperature × Flow	3	32	1.78	0.1708	0.143
FPD	Formulation × Flow	1	32	1.24	0.2732	0.037
FPD	Temperature × Formulation × Flow	3	32	7.90	0.00044	0.426
FPF	Temperature	3	32	4.91	0.0065	0.315
FPF	Formulation	1	32	4.90	0.0341	0.133
FPF	Flow	1	32	117.06	3.19×10^{-12}	0.785
FPF	Temperature × Formulation	3	32	26.54	8.23×10^{-9}	0.713
FPF	Temperature × Flow	3	32	10.39	6.29×10^{-5}	0.493
FPF	Formulation × Flow	1	32	5.82	0.0217	0.154
FPF	Temperature × Formulation × Flow	3	32	5.36	0.0042	0.334
Dose-normalised FPD	Temperature	3	32	6.75	0.0012	0.387
Dose-normalised FPD	Formulation	1	32	323.71	2.69×10^{-18}	0.910
Dose-normalised FPD	Flow	1	32	96.69	3.42×10^{-11}	0.751

Dose-normalised FPD	Temperature × Formulation	3	32	23.33	3.42×10^{-8}	0.686
Dose-normalised FPD	Temperature × Flow	3	32	19.62	2.12×10^{-7}	0.648
Dose-normalised FPD	Formulation × Flow	1	32	7.20	0.0114	0.184
Dose-normalised FPD	Temperature × Formulation × Flow	3	32	4.06	0.0149	0.276
Delivered Fraction	Temperature	3	32	2.785	0.0566	0.207
Delivered Fraction	Formulation	1	32	140.048	3.16×10^{-13}	0.814
Delivered Fraction	Flow	1	32	115.604	3.74×10^{-12}	0.783
Delivered Fraction	Temperature × Formulation	3	32	3.729	0.0209	0.259
Delivered Fraction	Temperature × Flow	3	32	2.755	0.0585	0.205
Delivered Fraction	Formulation × Flow	1	32	1.765	0.1934	0.052
Delivered Fraction	Temperature × Formulation × Flow	3	32	1.997	0.1342	0.158

The (Response ~ Temperature × Formulation × Flow). Partial η^2 values represent effect sizes

S8. Welch Pairwise Contrasts Table

Endpoint	Temp	Flow	Mean Difference (Isotonic – Hypertonic)	95% CI_low	95% CI_high	P_value
MMAD (μm)	5	30	-0.419	-0.639	-0.199	0.014
MMAD (μm)	5	50	-0.841	-1.244	-0.438	0.011
MMAD (μm)	21	30	-0.457	-0.850	-0.064	0.033
MMAD (μm)	21	50	-0.285	-0.362	-0.208	0.001
MMAD (μm)	37	30	0.087	-0.137	0.310	0.331
MMAD (μm)	37	50	-0.210	-0.727	0.307	0.232
MMAD (μm)	40	30	0.207	0.088	0.326	0.012
MMAD (μm)	40	50	-0.144	-0.256	-0.031	0.025
GSD	5	30	0.008	-0.020	0.036	0.462
GSD	5	50	0.199	0.117	0.280	0.003
GSD	21	30	-0.042	-0.247	0.163	0.557
GSD	21	50	-0.071	-0.126	-0.015	0.024
GSD	37	30	0.025	-0.202	0.253	0.684
GSD	37	50	-0.108	-0.197	-0.019	0.029
GSD	40	30	-0.057	-0.170	0.057	0.190
GSD	40	50	-0.176	-0.327	-0.025	0.033
D16 (μm)	5	30	-0.153	-0.230	-0.076	0.012
D16 (μm)	5	50	-0.564	-0.688	-0.440	<0.001
D16 (μm)	21	30	-0.183	-0.377	0.011	0.057
D16 (μm)	21	50	-0.042	-0.123	0.039	0.220
D16 (μm)	37	30	-0.034	-0.294	0.227	0.687
D16 (μm)	37	50	-0.158	-0.428	0.111	0.142
D16 (μm)	40	30	0.155	0.069	0.241	0.008
D16 (μm)	40	50	-0.042	-0.090	0.006	0.070
D84 (μm)	5	30	-0.396	-0.552	-0.240	0.003
D84 (μm)	5	50	-1.127	-1.331	-0.924	0.001
D84 (μm)	21	30	-0.761	-2.071	0.549	0.178

D84 (μm)	21	50	-1.005	-1.098	-0.912	<0.001
D84 (μm)	37	30	0.014	-0.343	0.370	0.915
D84 (μm)	37	50	-0.181	-1.152	0.791	0.515
D84 (μm)	40	30	0.289	0.120	0.459	0.009
D84 (μm)	40	50	-0.537	-0.992	-0.082	0.031
FPF (%)	5	30	10.051	5.958	14.144	0.004
FPF (%)	5	50	5.636	-3.269	14.541	0.122
FPF (%)	21	30	6.636	-8.514	21.785	0.219
FPF (%)	21	50	8.228	0.434	16.022	0.045
FPF (%)	37	30	-0.050	-3.661	3.560	0.971
FPF (%)	37	50	-2.595	-4.648	-0.543	0.026
FPF (%)	40	30	-7.915	-12.463	-3.368	0.008
FPF (%)	40	50	-3.927	-8.480	0.627	0.066
Dose-normalised FPD (%)	5	30	17.539	15.171	19.908	0.001
Dose-normalised FPD (%)	5	50	22.060	13.400	30.720	0.003
Dose-normalised FPD (%)	21	30	14.831	7.260	22.402	0.007
Dose-normalised FPD (%)	21	50	26.312	18.916	33.707	0.001
Dose-normalised FPD (%)	37	30	9.734	4.964	14.503	0.013
Dose-normalised FPD (%)	37	50	6.231	2.223	10.238	0.020
Dose-normalised FPD (%)	40	30	5.581	-3.038	14.200	0.117
Dose-normalised FPD (%)	40	50	8.054	-1.256	17.364	0.068
Delivered Fraction (%)	5	30	15.998	13.196	18.801	<0.001
Delivered Fraction (%)	5	50	20.891	9.978	31.805	0.012
Delivered Fraction (%)	21	30	11.437	-14.156	37.030	0.203
Delivered Fraction (%)	21	50	23.791	17.357	30.225	0.001
Delivered Fraction (%)	37	30	10.833	5.504	16.161	0.006
Delivered Fraction (%)	37	50	7.776	3.386	12.166	0.013
Delivered Fraction (%)	40	30	11.758	-3.570	27.086	0.081
Delivered Fraction (%)	40	50	10.191	-2.105	22.487	0.073

S9. Model Diagnostics

Model assumptions were evaluated using Shapiro-Wilk tests of residual normality and Levene's tests of homogeneity of variance. Homogeneity of variance was supported for all analysed endpoints (Levene's test $p > 0.05$). Several endpoints exhibited departures from strict residual normality according to Shapiro-Wilk testing. However, the study employed a fully balanced factorial design with equal replication across treatment combinations, and the principal findings were characterised by large effect sizes and consistent response patterns across multiple related endpoints. Consequently, these departures were not considered likely to materially affect interpretation of the ANOVA results, and the ANOVA framework was retained for inference.

Table S5. Model Diagnostics for Primary Endpoints.

Metric	Shapiro-Wilk p-value	Levene's Test p-value
MMAD	0.0211	0.7278
GSD	0.3737	0.7075
D16	0.0151	0.7018
D84	0.0001	0.2410
FPF	0.3682	0.7031
Dose-normalised FPD	0.8753	0.8648
Delivered Fraction	0.0021	0.6539

S10. False Discovery Rate Sensitivity Analysis

A Benjamini-Hochberg false discovery rate correction was applied across all matched ISS-versus-3% HSS Welch comparisons. Of the comparisons that were significant at the unadjusted level, all but two retained significance following FDR correction ($q<0.05$). Only one of the two comparisons corresponded to an endpoint reported in the manuscript: FPF at 21 °C and 50 L·min^{-1}, which was nominally significant before adjustment ($p=0.0451$) but did not remain significant after FDR correction ($q=0.0657$). The second comparison involved FPF Recovered, an additional exploratory analytical endpoint that was not used in interpreting the primary study findings. The FDR sensitivity analysis did not alter the primary factorial findings or the principal conclusions of the study.

Table S6. Manuscript Endpoint That Did Not Retain Significance Following FDR Correction.

Endpoint	Temperature (°C)	Flow (L·min^{-1})	Raw p-value	FDR adjusted q-value
FPF (%)	21	50	0.0451	0.0657

S11. Regional and Individual NGI-Stage Deposition Expressed as Percentage of Nominal Loaded Dose

ISS- 30LPM

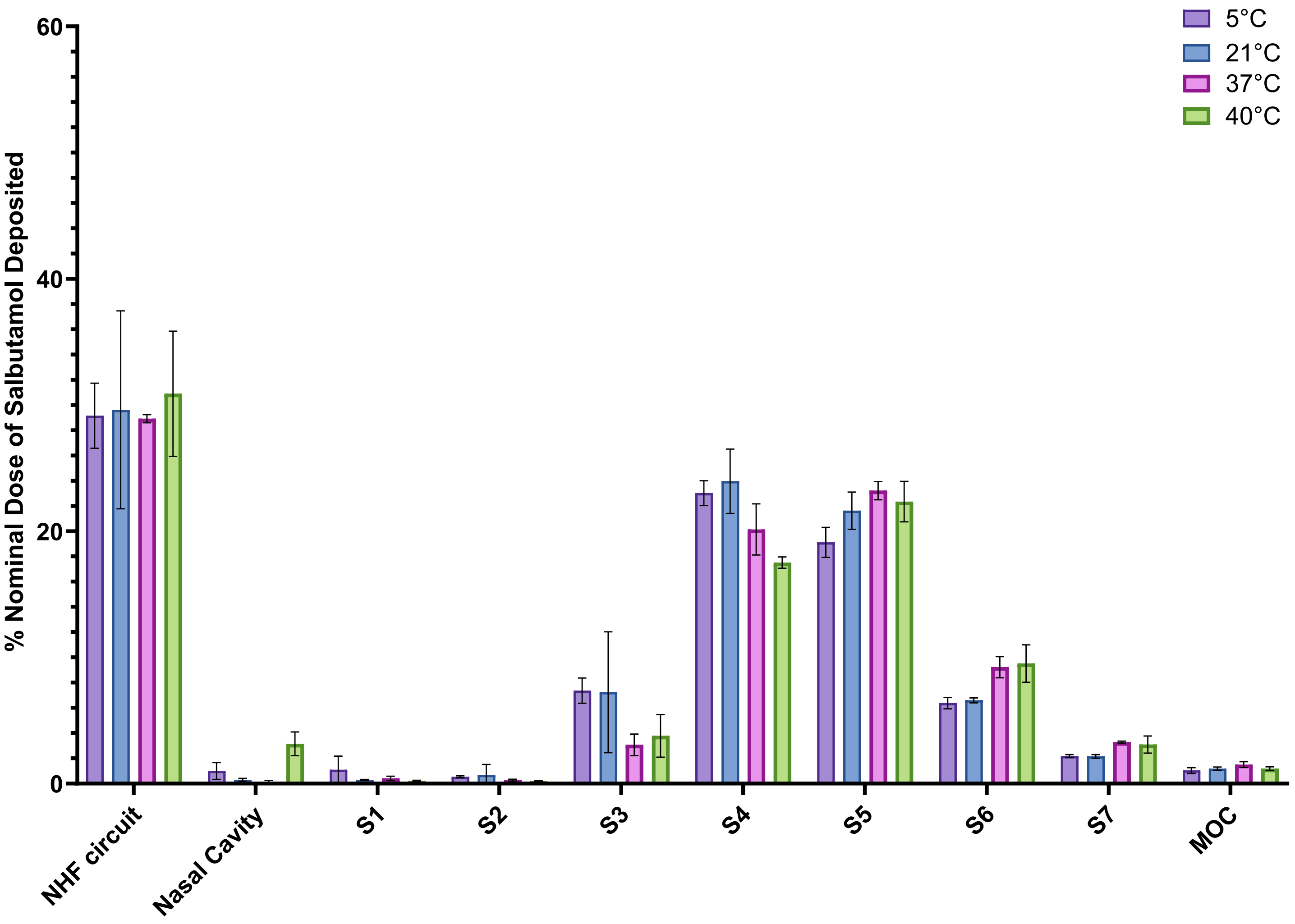

ISS-50LPM

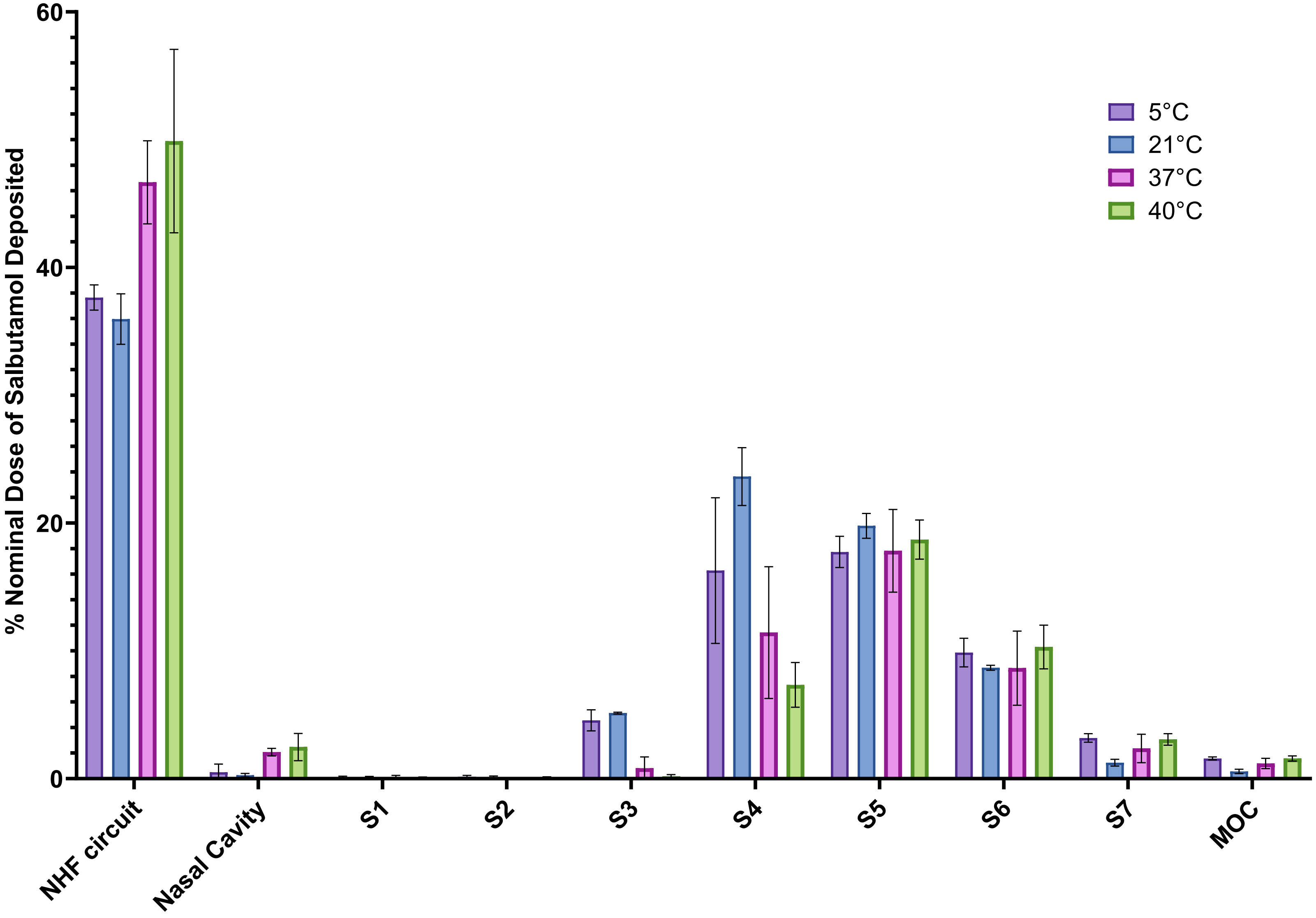

% Nominal Dose of Salbutamol Deposited
60
40
20
0
5°C
21°C
37°C
40°C
NHF circuit
Nasal Cavity
S1
S2
S3
S4
S5
S6
S7
MOC

3 % HSS 30LPM

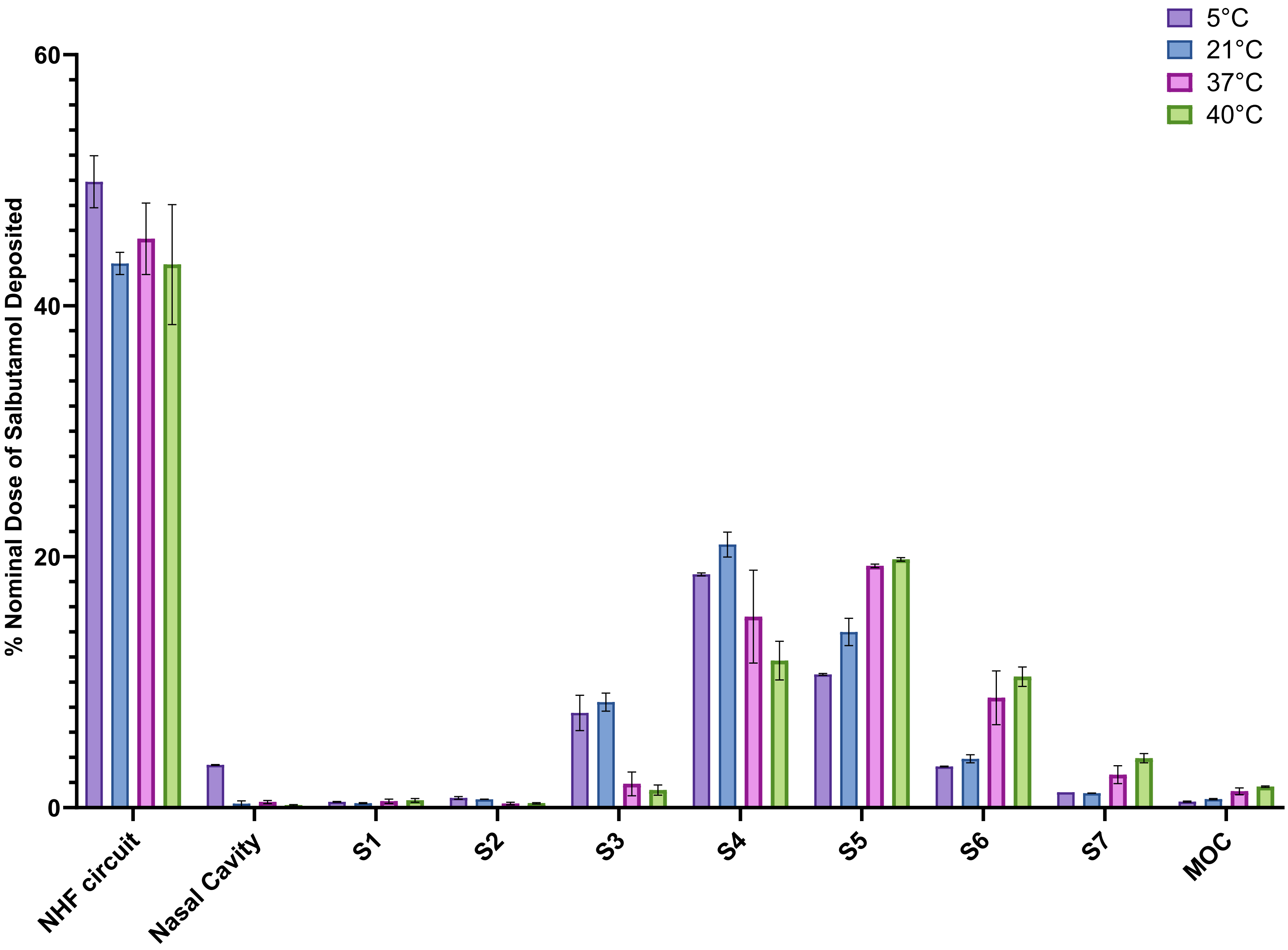

5°C
21°C
37°C
40°C
60
40
20
0
% Nominal Dose of Salbutamol Deposited
NHF circuit
Nasal Cavity
S1
S2
S3
S4
S5
S6
S7
MOC

3 %HSS 50LPM

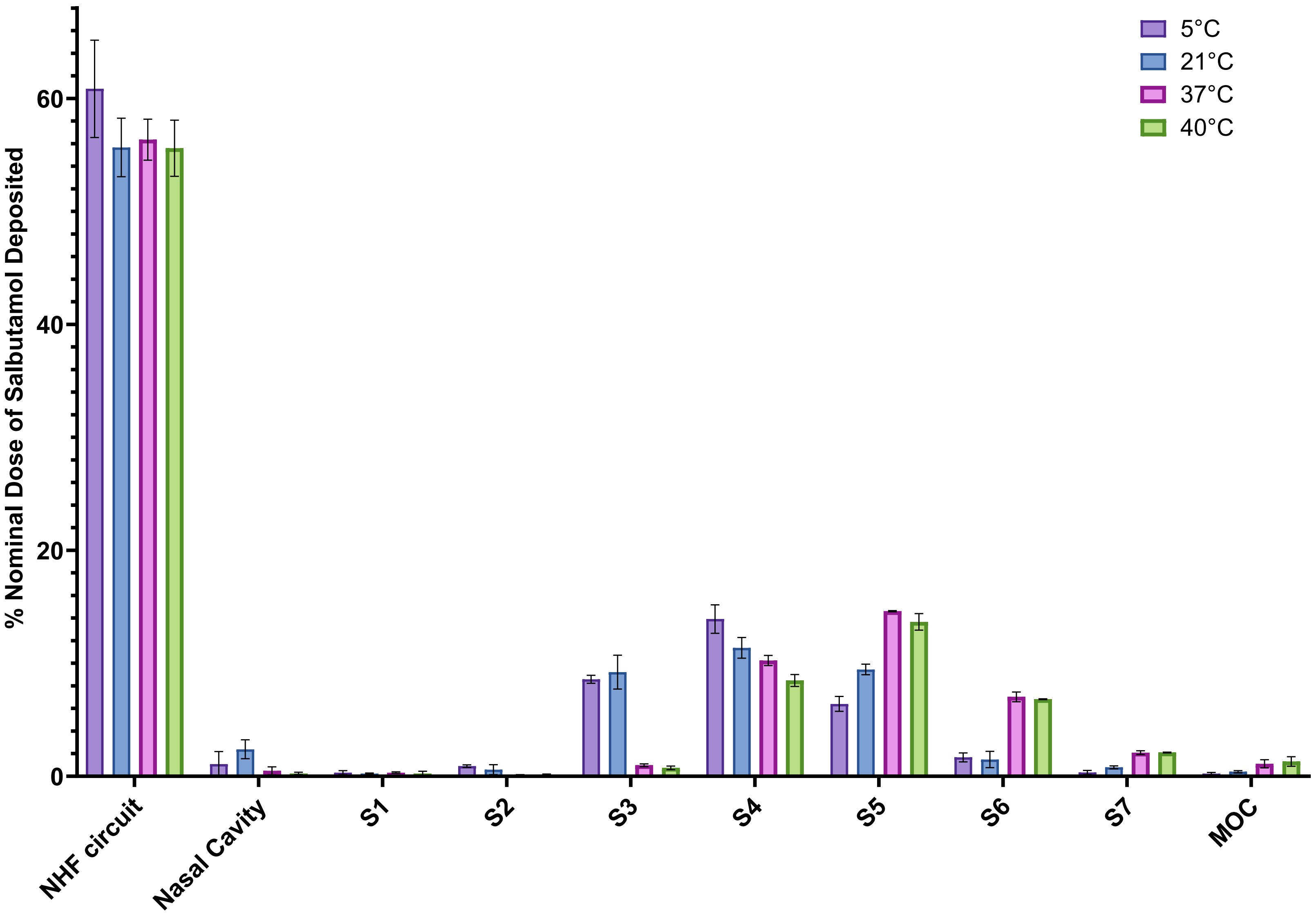

% Nominal Dose of Salbutamol Deposited
0
20
40
60
5°C
21°C
37°C
40°C
NHF circuit
Nasal Cavity
S1
S2
S3
S4
S5
S6
S7
MOC

S12. Pre-Device Drug Loss

Pre-device drug loss represents drug mass lost before aerosol entry into the delivery device, attributable to residual material remaining in the ampoule during formulation transfer. This loss occurs prior to aerosol generation and is therefore mechanistically distinct from device- or pathway-related aerosol losses.

- Commercial isotonic formulation: calculated as above
- 3 % hypertonic formulation**:** 0 % by design (direct pipette transfer; no ampoule)

Pre-device drug loss was calculated for each isotonic run as the fraction of nominal loaded dose recovered from the ampoule after transfer. For the hypertonic formulation, which was transferred directly into the nebuliser by pipette, pre-device drug loss was zero by design. Pre-device drug loss was averaged across replicates for each temperature–flow condition and reported as mean ± SD. This term was excluded from NHF circuit deposition and regional deposition analyses, which were normalised to recovered aerosol mass only.

System temperature (°C)	Flow (L·min^{-1})	Pre-device drug loss (%)
5	30	1.08 ± 0.72
5	50	0.68 ± 0.01
21	30	0.38 ± 0.11
21	50	0.30 ± 0.16
37	30	0.57 ± 0.26
37	50	0.48 ± 0.13
40	30	1.36 ± 1.06
40	50	0.36 ± 0.22
